# First core microsatellite panel identification in Apennine brown bears (*Ursus arctos marsicanus*): a collaborative approach

**DOI:** 10.1186/s12864-021-07915-5

**Published:** 2021-08-18

**Authors:** Erminia Scarpulla, Alessio Boattini, Mario Cozzo, Patrizia Giangregorio, Paolo Ciucci, Nadia Mucci, Ettore Randi, Francesca Davoli

**Affiliations:** 1 Department of Biological, Geological and Environmental Sciences (BiGeA), University of Bologna, Bologna, Italy; 2 Department for the Monitoring and Protection of the Environment and for Biodiversity Conservation, Unit for Conservation Genetics (BIO-CGE), Italian Institute for Environmental Protection and Research (ISPRA), Via Ca’ Fornacetta, 9 - 40064 Ozzano dell’Emilia, Bologna, Italy; 3 Department of Biology and Biotechnology “Charles Darwin” (BBCD), Sapienza University of Rome, Rome, Italy; 4 Faculty of Engineering and Science, Department of Chemistry and Bioscience, University of Aalborg, Aalborg, Denmark

**Keywords:** Conservation genetics, Cross-species amplification, Discriminatory power, Individual identification, Interlaboratory comparison, Italy, Non-invasive genetic profiles, Reproducibility, STR calibration

## Abstract

**Background:**

The low cost and rapidity of microsatellite analysis have led to the development of several markers for many species. Because in non-invasive genetics it is recommended to genotype individuals using few loci, generally a subset of markers is selected. The choice of different marker panels by different research groups studying the same population can cause problems and bias in data analysis. A priority issue in conservation genetics is the comparability of data produced by different labs with different methods. Here, we compared data from previous and ongoing studies to identify a panel of microsatellite loci efficient for the long-term monitoring of Apennine brown bears (*Ursus arctos marsicanus*), aiming at reducing genotyping uncertainty and allowing reliable individual identifications overtimes.

**Results:**

We examined all microsatellite markers used up to now and identified 19 candidate loci. We evaluated the efficacy of 13 of the most commonly used loci analyzing 194 DNA samples belonging to 113 distinct bears selected from the Italian national biobank. We compared data from 4 different marker subsets on the basis of genotyping errors, allelic patterns, observed and expected heterozygosity, discriminatory powers, number of mismatching pairs, and probability of identity. The optimal marker set was selected evaluating the low molecular weight, the high discriminatory power, and the low occurrence of genotyping errors of each primer. We calibrated allele calls and verified matches among genotypes obtained in previous studies using the complete set of 13 STRs (Short Tandem Repeats), analyzing six invasive DNA samples from distinct individuals. Differences in allele-sizing between labs were consistent, showing a substantial overlap of the individual genotyping.

**Conclusions:**

The proposed marker set comprises 11 *Ursus* specific markers with the addition of cxx20, the canid-locus less prone to genotyping errors, in order to prevent underestimation (maximizing the discriminatory power) and overestimation (minimizing the genotyping errors) of the number of Apennine brown bears. The selected markers allow saving time and costs with the amplification in multiplex of all loci thanks to the same annealing temperature. Our work optimizes the available resources by identifying a shared panel and a uniform methodology capable of improving comparisons between past and future studies.

**Supplementary Information:**

The online version contains supplementary material available at 10.1186/s12864-021-07915-5.

## Background

The loss of genetic variability is one of the main factors affecting the chance of species and populations to adapt in response to climate and ecological changes. Its negative consequences are particularly feared in small and isolated populations that present a level of inbreeding risk and fixation of deleterious alleles higher than in large and widespread populations [1]. During the last century, large mammals have been affected by habitat reduction that led to a contraction of population ranges and loss of random mating with a higher incidence of inbreeding risk. Large mammals, particularly those, like the brown bear, living in mountainous and forested areas, are always difficult to observe: their crepuscular and nocturnal habits, low density, large home ranges, and elusive behavior make it difficult and expensive to use traditional field census methods [2–6]. During the last decades, a variety of non-invasive sampling methods has become available for the demographic study of species with low detectability [7–9]. Among these, non-invasive genetic sampling (NIGS) largely prevails [10–16]. NIGS does not require live-trapping and individual marking, thus reducing behavioral reactions or injuries to the animals, and has been widely used to address important issues in species behavioral ecology and genetics [17–20]. Powerful individual identification from NIGS, through genotyping of hypervariable molecular markers (e.g. STRs), have been used to obtain useful information on population size, genetic variability, and population dynamics [20–24]. Hypervariable microsatellites (short tandem repeats; STR) are adequate to study genetically depauperate populations [25–28] and they can be amplified from low quality DNA, often provided by non-invasive samples. Analyzing many loci is expensive and increases the risk of errors in individual genotyping [29–31], a fact that often brings researchers to select a limited number of loci. It is known that the choice of markers affects the estimates of genetic diversity, so it is possible that by using fewer and not shared loci, research groups may produce different results, limiting the reproducibility of individual studies and reaching erroneous conclusions in comparative studies [32]. Although they are rarely considered, efforts to identify shared markers sets among different research groups studying the same species are of particular importance in limiting these kinds of errors [33, 34]. Careful selection of markers is critical to obtaining accurate and reproducible estimates of population structure, genetic diversity, or individual assignment [35] and can minimize potential bias by ensuring that inferred genetic patterns are comparable among studies. The accuracy of DNA-based identifications, obtained by limited numbers of STR, can be improved by using quality-control protocols (QC) to detect and eliminate genotyping errors [3, 36, 37].

The dynamics of population size and density are critical in the decision-making processes for the management and conservation of animal populations [38]. They play a key role in determining genetic diversity, susceptibility to stochastic mortality factors, and ultimately population extinction risk [39]. For these reasons, genetic monitoring of the wild populations can be used to document any change in genetic variability and diversity, but only when protocols can be compared. The application of different approaches, such as the use of different STR panels, hampers the opportunity to combine results over time and prevents any chance of obtaining reliable and useful information.

The Apennine brown bear (*Ursus arctos marsicanus*; ABB) is a small relict brown bear population living in complete geographic isolation in the central Italian Apennines [40, 41], whose conservation status is critical [42]. Based on morphological [43, 44] genetic [28] and genomic [45] evidence ABB is considered an evolutionary and management unit, deserving particular conservation attention. Because of historical habitat contraction and persistent anthropogenic mortality [40, 46], the population is highly depleted of genetic variability. Therefore, the action plan for the conservation of ABB (PATOM [47]) highlighted the urgent need to produce reliable population estimates and to conduct long-term monitoring of the population size to assess its trends and evaluate the effectiveness of conservation strategies.

The endangered ABB population has been monitored by the Italian Forest Service using NIGS since 1991, first collecting samples with opportunistic methods, then, from 2001 until now, by hair-snag techniques [48]. The monitoring project aimed to assess of the ABB minimum population size [49]. The first formal estimate of population size (*N* = 40 bears in the core area; 95% CI = 37–52) was obtained in 2008 using capture-mark-recapture models (CMR) of genotypes [50]. The same method was applied in the European Life+ NAT/IT/000160 project (Life Arctos), producing an estimate of 51 bears (95% CI: 47–66) including cubs in 2011 [51] and of 50 bears (95% CI: 45–69) in 2014 [52].

In conservation genetics, it is crucial to promote the comparability of data across different labs based on standardized methods, to allow the implementation of a shared database to be used in conservation actions and to obtain estimates of population size using capture-mark-recapture models (CMR) of genotypes. Two problems can frustrate individual identification through DNA fingerprinting: (i) first, when not enough loci are examined or the loci do not have high enough discriminatory power, different individuals with the same genotype will be indistinguishable leading to an underestimation of the number of individuals (“shadow effect”); (ii) second, genotyping errors can determine differences in different samples coming from the same individual leading to an over estimation of the number of individuals. Moreover, considering the extremely low genetic variability of the ABB population, it is important to establish a reliable and shared set of markers for the robust individual identification in long-term monitoring projects.

**Fig. 1:**
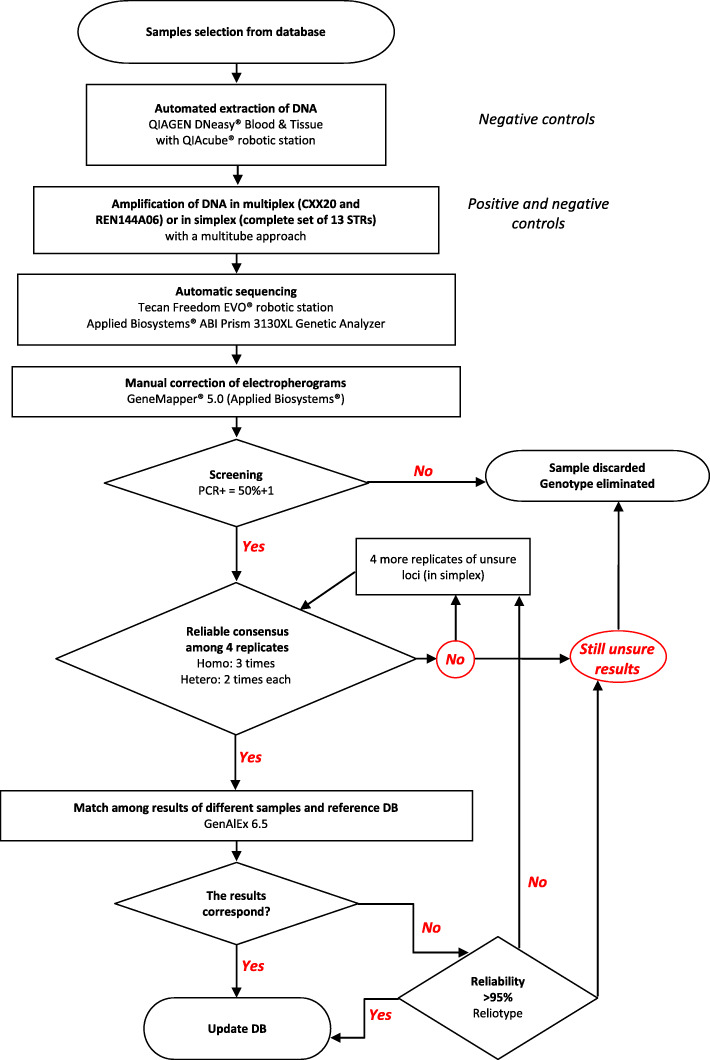
Flow chart diagram illustrating the QC procedure used to obtain a reliable genotyping. Process showing how to obtain genotypes with a confidence level of 95% (RelioType [53])

We chose to test 4 subsets of loci in order to: (i) maximize our ability to compare results across studies, (ii) minimize the costs of analysis and the risks of genotyping errors, (iii) identify a set of markers to allow long-term monitoring of the Apennine brown bear population. Our goals were to identify a core STR panel that can be utilized by different labs for a variety of conservation and management purposes (e.g. population genetics, individual assignment, parentage analysis) and to detail a uniform methodology in order to improve repeatability and comparative efforts in the long-term monitoring projects. The genotyping protocol for ABB non-invasive samples was developed aiming to: (i) determine the optimal set of STR markers for individual identification avoiding the risk of misidentification; and (ii) establish reliable genotyping procedures to minimize the shadow effect overtime. The proposed panel was evaluated based on efficacy and interpretability and the reliability of the protocol has been assessed by QC procedure (Fig. 1), and used to reconstruct all the ABB genotypes obtained so far.

## Results

### Review of microsatellite and panel selection

We reviewed 7 peer-reviewed papers that used microsatellite loci to study aspects of ABB genetics published between 2004 and 2017 (Table 1). The most common application was the estimate of the population size (4) followed by individual identification (2) and estimate survival (1).

**Table 1:** Summary of STR loci used in previous studies of Apennine brown bear genetics

Date	Citation	Application	Lab	Sex	American black bears loci										European brown bears loci						Asiatic black bears locus	Canid loci		Total
				AMG	G1 A	G1 D	G10 B	G10 C	G10 H	G10 J	G10 L	G10 M	G10 P	G10 X	Mu 05	Mu 11	Mu 15	Mu 50	Mu 51	Mu 59	MSUT-2	Cxx 20	REN 144A06	
2004	Lorenzini et al. (Animal Conservation)	Individual identification	Experimental Zooprophylactic Institute of Abruzzo and Molise G. Caporale” (IZSAM) – Lab1		x	**x**	**x**	**x**	x	x	**x**	x	**x**	x	**x**			**x**		**x**		**x**		14
2008	Gervasi et al. (Ursus)	Estimate population size	ISPRA BIO-CGE (ex-INFS) – Lab2	**x**			**x**	**x**			**x**				**x**	**x**	**x**	**x**	**x**	**x**				9
2010	Gervasi et al. (Conservation Genetics)	Estimate population size	ISPRA BIO-CGE (ex-INFS) – Lab2	**x**			**x**	**x**			**x**				**x**	**x**	**x**	**x**	**x**	**x**				9
2012	Gervasi et al. (Biological Conservation)	Estimate population size	ISPRA BIO-CGE (ex-INFS) – Lab2	**x**		**x**	**x**	**x**			**x**		**x**		**x**	**x**	**x**	**x**	**x**	**x**				11
2014	Forconi et al. (Hystrix)	Individual identification	ISPRA BIO-CGE (ex-INFS) – Lab2	**x**			**x**	**x**			**x**				**x**	**x**	**x**	**x**	**x**	**x**				
2015	Ciucci et al. (Journal of Mammalogy)	Estimate population size	Wildlife Genetics International (WGI) – Lab3	**x**		**x**	**x**	**x**			**x**		**x**	x	**x**	**x**		**x**	**x**	**x**	x	**x**	**x**	14
2017	Gervasi et al. (Population Ecology)	Estimating survival	ISPRA BIO-CGE (ex-INFS) & Wildlife Genetics International (WGI) – Lab2 & Lab3	**x**		**x**	**x**	**x**			**x**		**x**		**x**	**x**		**x**	**x**	**x**				10
Total				**6**	1	**4**	**7**	**7**	1	1	**7**	1	**4**	2	**7**	**6**	**4**	**7**	**6**	**7**	1	**2**	**1**	19

Labs and applications are indicated. Loci included in the final microsatellite panel are in bold (see text for details)

What emerges from the literature review is that the individual brown bears genotyping was performed by three different labs: until 2002 by the Experimental Zooprophylactic Institute of Abruzzo and Molise “G. Caporale” (IZSAM - Lab1, Method 1 described in Lorenzini et al. [49]); in 2011 and 2014 by the Wildlife Genetics International lab (WGI - Lab2, Method 2 described in Ciucci et al. [52]); and since 2002 until now, except for the core area in 2011 and 2014, by the Unit for Conservation Genetics of the Italian Institute for Environmental Protection and Research (ISPRA BIO-CGE - Lab3, Method 3 described in Gervasi et al. [14, 41, 50] and Forconi et al. [54]). The most recent study [55] begins to address the problem of how to merge datasets between Lab2/Method 2 and Lab3/Method 3.

A total of 19 autosomal STR loci are reported from the reviewed papers, 7 of which (G1D, G10B, G10C, G10L, Mu05, Mu50, Mu59) were shared among Method 1, Method 2, and Method 3, and 9 (G1D, G10B, G10C, G10L, Mu05, Mu11, Mu50, Mu51, Mu59) were shared between Method 2 and Method 3 (plus G10P, used at a later stage of analysis at Lab2 to better discriminate equivocal cases [52], and the Y-linked Amelogenin gene AMG, used for sex determination). In addition to the other STRs, Lab2 also used two markers originally identified on canids (cxx20 and REN144A06).

Given the revision of the literature, in this study we identified 4 sets of STRs to compare: (i) 11 Lab2 loci, (ii) 11 Lab3 loci, (iii) 9 shared loci, and (iiii) the complete set of 13 loci (Additional file 2: Table S2): 9 shared loci between Lab2 and Lab3, plus cxx20 and REN144A06 used in the Method 2, plus G10P and Mu15 used in the Method 3. The complete set of 13 STRs comprises: 2 loci designed on canid genome (REN144A06 [56] and cxx20 [57]); 5 loci designed on a genomic library of American black bears (*Ursus americanus*, G10B, G10C, G10L, G10P and G1D [58, 59]); 6 loci designed on a genomic library of European brown bears (*Ursus arctos*, Mu05, Mu11, Mu15, Mu50, Mu51 and Mu59 [21]) (Additional file 3: Table S3).

### Inter-laboratory calibration

The calibration keys based on six invasive DNA samples, for the 13 STR included in this study, plus Amelogenin gene, are shown in Table 2. We compared 45 historic genotypes of individuals, that overlapped between Labs 2 and 3. Differences in allele-sizing between labs were consistent, showing a substantial overlap of the individual genotyping, and the determination of calibrations factors was possible (Table 2). However, two pairs of genotypes did not match at all loci (0.9% of mismatch between databases, Additional file 4: Table S4). Both pairs (ram0587-Gen 108 and HS374-Gen 105) mismatched at either cxx20 and REN144A06 (Additional file 4: Table S4). In the first pair, cxx20 and REN144A06 showed different alleles, therefore ram0587 and Gen 108 could be different individuals. In the second pair, cxx20 and REN144A06 were homozygotes in the Lab2, and heterozygotes in the Lab3. The remaining 43 pairs of genotypes yielded perfect matches at all 13 loci plus Amelogenin gene.

**Table 2:** Allelic ranges of the analyzed loci in the ABB population and their conversion factors

Locus	Allele size range (bp) Lab2^a^	Allele size range (bp) Lab3^b^	Calibration key Lab2/Lab3
CXX20	135–139	132–136	−3
REN144A06	109–129	110–130	+ 1
G1D	172–186	100–114	−72
Mu51	206–214	114–122	−92
G10B	140–156	112–128	−28
G10C	197–207	95–105	− 102
Mu59	229–235	101–107	−128
Mu11	188–196	88–96	−100
Mu05	135–137	135–137	–
G10L	157–163	148–154	−9
Mu50	132–136	100–104	−32
G10P	159–171	152–164	+ 7
Mu15	–	117–121	Not used at Lab2
Amelogenin	204–250	158–212	−46/−38

Factors of conversion are based on 6 invasive samples shared between Lab2 and Lab3, representing the amount to add or subtract at Lab2 scores to obtain Lab3 scores. ^a^ Ciucci et al. 2015, ^b^ Gervasi et al. 2008, 2010, 2012

### Marker polymorphism and power

To merge different datasets (with 9 loci in common) into a single database consisting of 13 loci, we selected from the Italian national biobank a total of 194 DNA samples (33 from blood, 8 from tissue, 146 from hair and 7 from feces, see Additional file 5: Table S5) belonging to 115 genotypes out of 125 unique genotypes (Additional file 5: Table S5): extracted DNA or biological samples were not available for a total of 10 bears out of 125, therefore it was not possible to update the genotypes belonging to these 10 bears with cxx20 and REN144A06. We genotyped a total of 115 individual brown bears (56 females, 57 males, 2 indeterminate) at 13 microsatellite loci, plus the Amelogenin sex marker.

Two genotypes were eliminated: Gen 98 (male), because it matched with Gen 99 (female) at all loci except for sexing, and Gen 53 (female) because it matched at all but one locus with Gen 43 (female) and it showed a missing data at locus G1D. Both genotypes Gen 98 and Gen 53 were sampled only twice with non invasive methods and were considered unsure, conversely, Gen 99 and Gen 43 have been sampled many times and also with invasive methods, so they are bears certainly present in the population. All the analysis to evaluate the level of polymorphism and the discriminatory power of the marker subsets were conducted on the remaining 113 genotypes.

All the tested markers were polymorphic and diversity indexes were calculated on the complete set of 13 markers (Table 3). An average of 2.5 ± 0.14 alleles per primer pair was detected, ranging from 2 to 3 alleles per locus. The number of effective alleles per locus varied from 2.6 in cxx20 to 1.2 in Mu15 and G10P, with an average of 1.96 ± 0.11. Shannon information index ranged from 0.31 to 1.02. The values of observed and expected heterozygosity do not differ significantly so the population can be considered in HWE (Hardy–Weinberg equilibrium). Microchecker 2.2.3 software suggested a possible presence of null alleles at locus Mu11 due to an excess of homozygosis. Furthermore, this locus shows a signal of deviation from HWE, albeit non-significant (Table 3).

**Table 3:** Genetic diversity parameters and genotyping errors of 13 STR markers evaluated in 113 bear genotypes

Locus	Multiplex	A	N_e_	H_o_	H_e_	I	HWE	P_ID_	P_IDsib_	ADO	FA
CXX20	3	3	2.6	0.655	0.619	1.02	ns	0.22	0.50	0.085	0.023
REN144A06	3	3	2.5	0.670	0.605	0.99	ns	0.24	0.51	0.076	0.039
G1D	2	3	2.3	0.670	0.573	0.95	ns	0.25	0.53	0	0
Mu51	1	3	2.2	0.554	0.560	0.94	ns	0.26	0.53	0.013	0
G10B	2	3	2.0	0.518	0.513	0.75	ns	0.36	0.58	0.159	0
G10C	1	3	2.0	0.509	0.501	0.71	ns	0.37	0.59	0	0
Mu59	2	2	1.9	0.554	0.488	0.68	ns	0.38	0.60	0	0
Mu11	1	3	1.8	0.333	0.456	0.67	*p* < 0.05	0.39	0.62	0.071	0
Mu05	1	2	1.8	0.500	0.459	0.65	ns	0.40	0.62	0.058	0
G10L	2	2	1.7	0.491	0.439	0.63	ns	0.41	0.63	0.052	0
Mu50	1	2	1.8	0.429	0.448	0.64	ns	0.41	0.63	0	0
G10P	1	2	1.2	0.180	0.207	0.36	ns	0.65	0.81	0.083	0
Mu15	2	2	1.2	0.188	0.170	0.31	ns	0.71	0.84	0	0.035

The table includes the number of PCR multiplexes, number of alleles (A), effective number of alleles (N_e_), expected (H_e_) and observed (H_o_) heterozygosity, Shannon information index (I), Hardy-Weinberg equilibrium (HWE), probability of identity for unrelated individuals (P_ID_) and for siblings (P_IDsib_), allelic dropouts (ADO), false alleles (FA). STR loci are listed from the most to the least informative

Canid loci (cxx20 and REN144A06) show higher frequencies of genotyping errors when compared to the ursid-specific loci (Table 3), namely a high occurrence of ADO (allelic dropouts, 0.085 for cxx20 and 0.076 for REN144A06) and FA (false alleles, 0.023 for cxx20 and 0.039 for REN144A06). The average values of genotyping errors of REN144A06 and cxx20 (ADO = 0.081; FA = 0.031) are much higher than the average values of the *Ursus* specific markers used for routine analysis (ADO = 0.048; FA = 0.003). Both genotyping errors show statistically significant differences when comparing the average values between canid and ursid loci (ADO *p* = 2.3 × 10^− 07^; FA *p* = 3.7 × 10^− 06^).

**Fig. 2:**
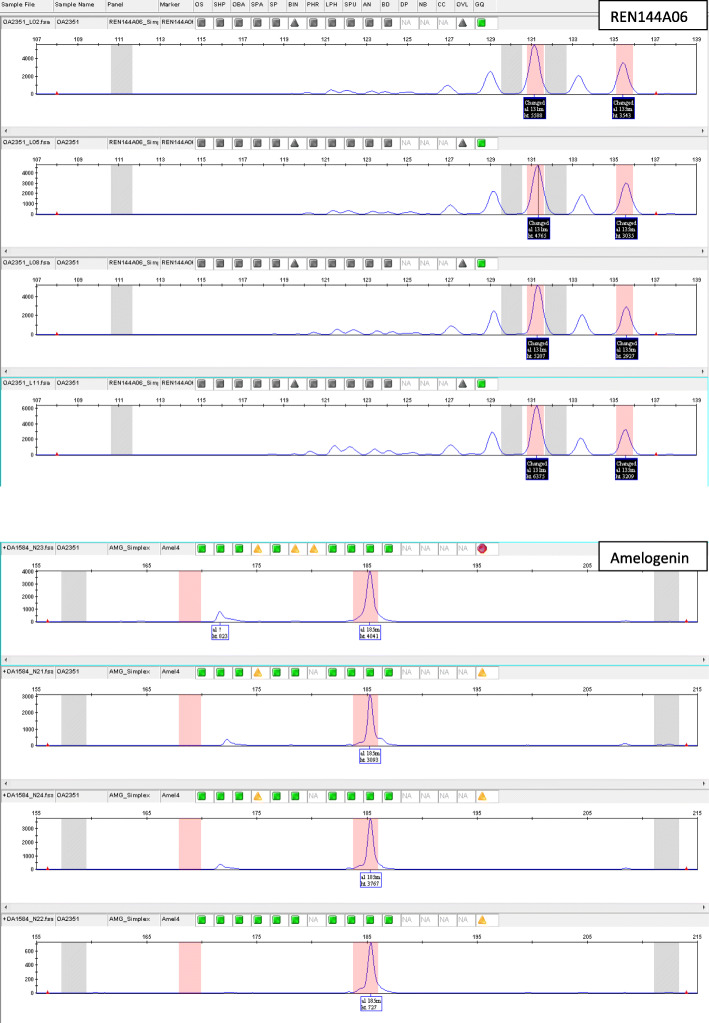
Electropherograms of REN144A06 and Amelogenin gene of a bear-canid mixed sample. Electropherograms refer to the non-invasive sample called OA2351 (Gen 84, see Additional file 5: Table S5 for the list of samples). Grey bands identify brown bear alleles, while pink bands identify canid alleles

Eight mixed samples were detected through the amplification of three alleles at both cxx20 and REN144A06. These samples can be mixed between different bears or between a bear and one or more canids. In addition, a bear sample (OA2351; Gen 84) mixed with canid DNA was detected through the amplification of different alleles than the known bear specific ones at both cxx20 and REN144A06 loci, confirmed by the results of the amplification of the Amelogenin gene (Fig. 2).

Observed and expected heterozygosity values did not show substantial differences and the error bands overlap among the 4 different STR sets (11 Lab2 loci, 11 Lab3 loci, 9 shared loci, and the complete set of 13 loci) indicating a non significance in the differences (Table 4).

**Table 4:** Estimates of genetic variability in the four different STR markers sets compared in this study

STRs set	N_e_	H_o_	H_e_	Pairs of genotypes/(113)^2^				P_ID_	P_IDsib_	P_ID_/100	P_IDsib_/100
				0 MM	1 MM	2 MM	3 MM				
9 shared STRs	1.98	0.508 ±0.030	0.493 ±0.016	2	21	75	267	8.5 × 10^−5^	8.9 × 10^−3^	0.009	0.890
11 STRs (Lab3)	1.85	0.449 ±0.046	0.438 ±0.039	0	10	56	168	3.9 × 10^−5^	6.1 × 10^−3^	0.004	0.610
11 STRs (Lab2)	2.09	0.535 ±0.031	0.514 ±0.019	1	4	17	71	4.5 × 10^−6^	2.3 × 10^− 3^	0.000	0.230
Complete set of 13 STRs	1.96	0.481 ±0.045	0.464 ±0.038	0	4	8	53	2.1 × 10^−6^	1.5 × 10^−3^	0.000	0.150

*N*_*e*_ number of effective alleles, *H*_*e*_ mean value of expected heterozygosity, *H*_*o*_ mean value of observed heterozygosity, *1–2-3MM* number of pairs of genotypes out of 12,769 pairs in total (113^2^) matching at all but 1–2-3 loci, *0MM* number of identical pairs of genotypes out of 12,769 pairs in total (113^2^), *P*_*ID*_ probability of identity for unrelated individuals, *P*_*IDsib*_ probability of identity for siblings, *P*_*ID*_*-P*_*IDsib*_*/100* number of bears in 100 that could show, by chance, the same multilocus genotype based on P_ID_ or P_IDsib_ values. STR marker sets are listed from the least to the most informative on the basis of P_ID_ and P_IDsib_ values. Amelogenin gene was not included in the analysis but was included in the individual genotyping, reducing the number of similar genotypes reported in P_ID_-P_IDsib_/100 columns

**Fig. 3:**
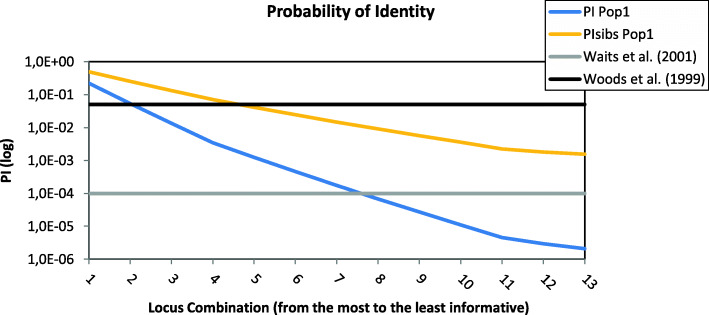
Probability of identity for unrelated individuals (P_ID_ in blue) and for siblings (P_IDsib_ in red). The P_ID_ threshold of < 0.001 suggested by Waits et al. [37] (in green) and the P_IDsib_ threshold of < 0.05 suggested by Woods et al. [20] (in purple) are included. Loci are added to the combinations in order from the most to the least informative

**Fig. 4:**
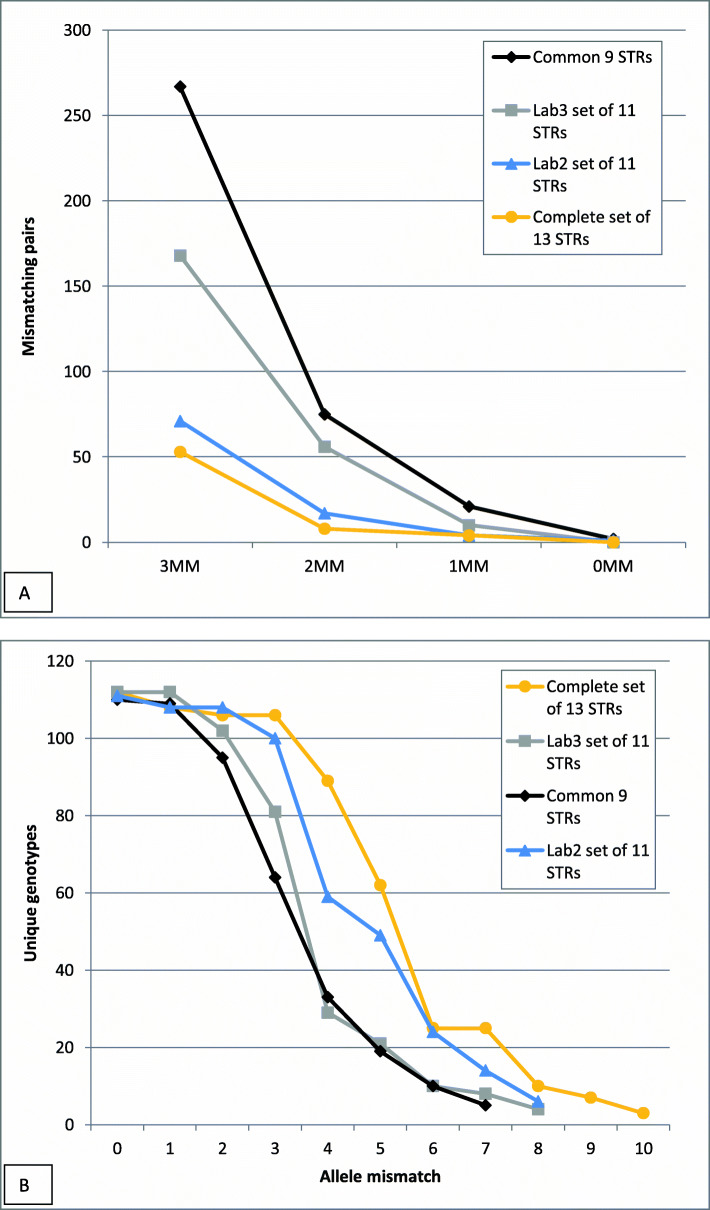
Mismatches distribution in Apennine brown bears population. The analysis was carried out on 113 Apennine brown bears based on the four different STR marker sets compared in this study. **A** Number of mismatching pairs and **B** unique genotypes for each STR marker set

The values of probability of identity (P_ID_) and probability of identity among siblings (P_IDsib_) decrease with the increasing of the locus combination (Fig. 3). P_ID_ values differ by one order of magnitude among marker sets, while P_IDsib_ did not decrease significantly (Table 4). The complete set of 13 STRs and the set of Method 3 had no pairs of genotypes with zero mismatches (0MM), but, when considering 1, 2 and 3MM, the complete set of 13 STRs and the set of Method 2 showed a lower number of mismatching pairs (Table 4, Fig. 4A).

The number of unique genotypes, corresponding to a specific mismatch value, that can occur in different STR sets (Fig. 4B), demonstrated an improvement, albeit not significant, of the discrimination power obtained using 13 STRs compared to the other STR sets.

### Trends in genetic diversity over time

**Fig. 5:**
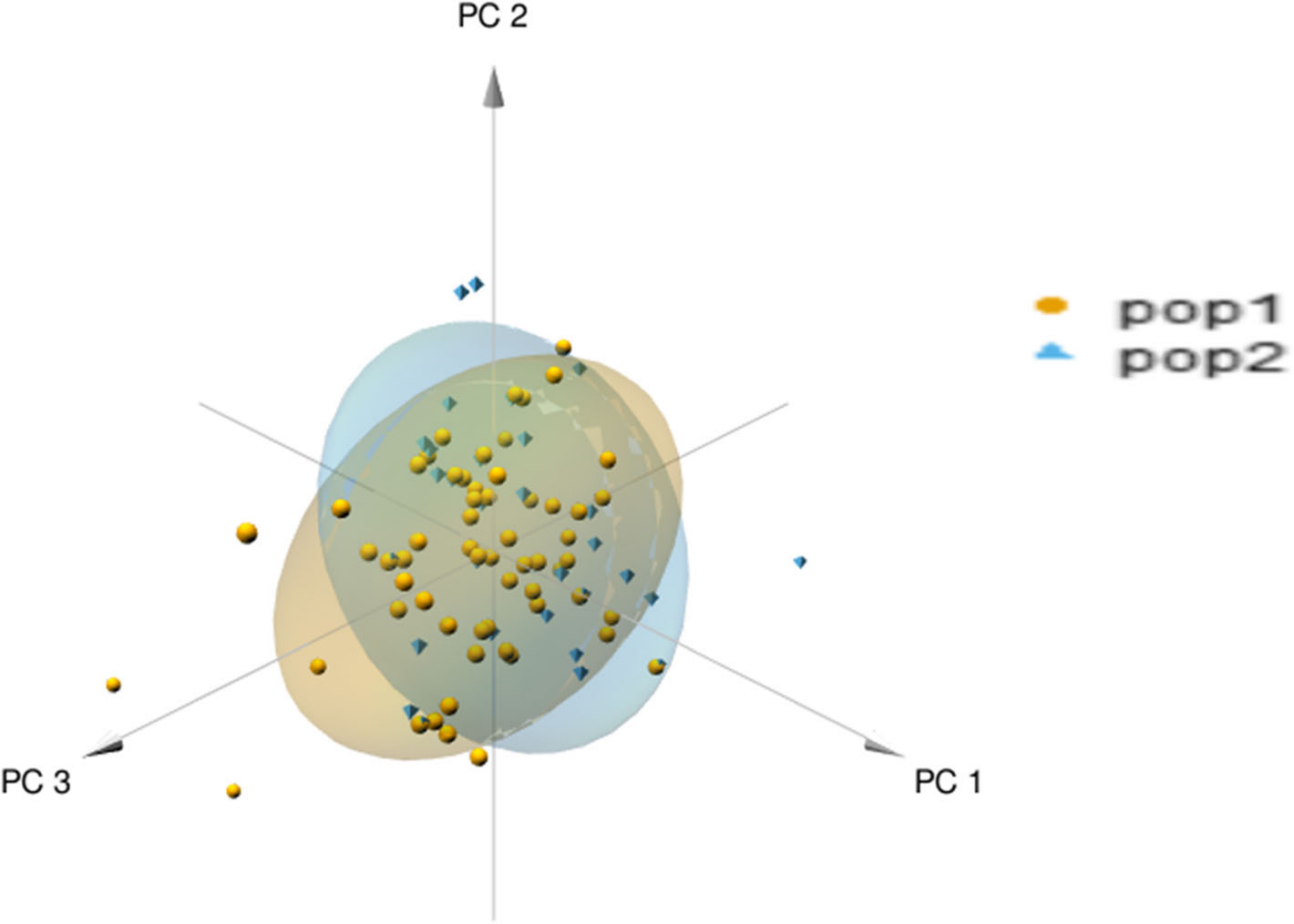
3D plot of principal component analysis of the observed genetic variation in Apennine brown bears. PCA was performed with 98 individuals and 13 STRs. pop1 = pre-arctos (2000–2010), pop2 = arctos&post (2011–2017)

To assess the resolution of the selected STR in describing the genetic status of the population over time, we grouped the genotypes in two periods (Additional file 6: Table S6), based on the years they were sampled: (i) pop1 - pre-arctos and (ii) pop2 - arctos & post-arctos. The allelic patterns show only slight variation over time, even with the elimination of 14 genotypes that were sampled in both periods, that could have flattened the differences (Additional file 8: Table S7). There are differences in allelic frequencies and some rare alleles seem to be lost from 2011 (126 at locus G10B; 95 at locus G10C), showing the most powerful effect of genetic drift (Additional file 8: Table S7). Nevertheless, the effective number of alleles goes from 1.96 ± 0.104 to 1.92 ± 0.125 showing a decrement of only 0.004. Also the number of private alleles is higher in the pre-arctos (0.15 ± 0.104) than in the arctos & post (0.07 ± 0.077) period. Expected heterozygosity decreases only from 0.47 ± 0.035 to 0.45 ± 0.039. As for F statistics, most loci show an excess of heterozygosis (Table 5), and the Fst index shows that the population has not significantly changed over the 18-year period. This is confirmed by the fact that Fis and Fit have almost the same value. The principal component analysis (PCA analysis, Fig. 5) shows a substantial overlap of genetic diversity in the two considered periods of time. Finally, the AMOVA test (Analysis of MOlecular VAriance) confirms that the variability is all concentrated within groups rather than among groups (F = − 0.003, *p* = 0.59).

**Table 5:** F-statistics (Fis, Fit, and Fst) per locus and mean values

Locus	F_is_	F_it_	F_st_
CXX20	−0.073	−0.063	0.009
REN144A06	−0.126	−0.125	0.001
G1D	−0.129	−0.129	0.000
Mu51	0.063	0.067	0.004
G10B	−0.028	−0.028	0.000
G10C	−0.042	−0.041	0.001
Mu59	−0.134	−0.125	0.008
Mu11	0.259	0.261	0.002
Mu05	−0.092	−0.090	0.001
G10L	− 0.118	− 0.113	0.004
Mu50	0.051	0.053	0.001
G10P	0.119	0.125	0.007
Mu15	−0.096	−0.096	0.000
Mean	−0.026 ± 0.033	− 0.023 ± 0.033	0.003 ± 0.001

Positive values of F_is_ and F_it_ indicate a deficiency of heterozygosis, while negative values indicate an excess

## Discussion

We have identified a microsatellite panel suitable for reliable individual identification of Apennine brown bears for long-term monitoring that is the set of *Ursus* specific markers with the addition of cxx20, the canid-locus less prone to genotyping errors.

This study showed that the Apennine brown bear population has an exceptionally low genetic variability (He = 0.46 ± 0.038). This level can be compared to the low expected heterozygosity of isolated brown bear populations in the Yellowstone Ecosystem (He = 0.55 [60]), in Baranof and Chichagof Islands in Alaska (He = 0.50 [17]), and in Cantabria – Spain, Western subpopulation (He = 0.45 [61]). By contrast, more connected and widespread populations have higher levels of heterozygosity, like the Alaskan (He = 0.78 [17]) and the Scandinavian brown bears (He = 0.70 [60]). Low levels of variability is a factor that endangers an already depleted population and increases the chance that more bears share the same multilocus genotype [62]. Therefore it is important to choose the best STR markers set, in order to reduce genotyping errors as much as possible and increase the chance of correctly identifying individuals. This issue was dealt with by comparing the different STR marker sets with various methods.

Our literature review produced a list of 19 STR loci used to assess different aspects of ABB demography and genetics during the last 20 years. A smaller proportion of these loci were used in previous studies on Apennine brown bear and represent appropriate candidates for a core microsatellite panel.

First of all, conversions factors are provided. The calibration among labs showed a substantial overlap of the compared STR datasets: only 0.9% of mismatch is present between databases. The mismatches observed among putative identical genotypes that differ between labs are mainly due to the canid loci. As expected, non ursid-specific canid loci have a higher rate of false alleles than ursidae loci and showed a high occurrence of ADO. Errors deriving from cross-amplification and contamination cannot be corrected because they show up in each replicate. Contamination can occur with exogenous DNA directly in the field, therefore it is important to use species-specific markers in order to avoid the amplification of alleles belonging to other species. Since only the non ursid-specific STRs show the amplification of three alleles, the most likely explanation is cross-contamination with other species. The detection of mixed samples is particularly challenging in species with a mean of 2–3 alleles per locus, since the most reliable indication of a mixed sample is the amplification of more than 2 alleles per locus. When a mixed sample is not detected due to low variability, it leads to the identification of chimera genotypes, that are not real individual genotypes but admixtures of different genotypes. If genotyping errors are not detected and corrected they lead to the assessment of false multilocus genotypes that ultimately causes the overestimation of the population size. However, the posterior quality control assures the reliability of the genotypes in the database, avoiding the presence of chimeras (e. g. Gen 98, and Gen 53). In addition, Cxx20 and REN144A06 introduce the risk of erroneous individual idenfitication, because of the possible amplification of alleles from exogenous DNA belonging to other species than *Ursus arctos marsicanus*.

Non-invasive sampling allows the identification of individuals through DNA fingerprinting techniques and it is the first step to capture-mark-recapture studies to assess the size of natural populations. The probability that two individuals in a population have the same multilocus genotype, is defined by the probability of identity (P_ID_), that depends on the number of analyzed loci, allelic diversity and percentage of related individuals in the population studied [37]. The P_ID_ is used more and more to assess the statistical confidence for individual identification [63, 64]. Waits et al. [37] evaluated the accuracy of a P_ID_ estimation by comparing the observed and expected P_ID_, using large nuclear DNA STR data sets from three endangered species (grey wolf, brown bear and the Australian northern hairy-nosed wombat). In some cases, the bias between the two indicators was three orders of magnitude, probably due to population substructure and the presence of close relatives in the dataset. For example, the tendency of bear cubs to remain with their mother for 15–17 months [65, 66] may result in sampling a large proportion of closely related individuals. In addition, past demographic events such as bottlenecks that caused a disruption in Hardy-Weinberg equilibrium may lead to inaccurate estimations with the expected P_ID_ index. In these cases, the standard P_ID_ estimator can lead to an underestimate of the minimum population size when too few nuclear DNA STR loci are used to identify individuals. Potential errors associated with estimating P_ID_ can be avoided by choosing the P_IDsib_ estimator as a conservative upper bound of the number of loci necessary to distinguish individuals. However, it is not simple to set an adequate threshold for P_ID_. Woods et al. [20] proposed a criterion of P_IDsib_ < 0.05 for brown bear population estimation when using multilocus genotypes amplified from hair samples. In wildlife forensic cases, P_ID_ < 0.001–0.0001 has been used, depending on the size of the source population [37]. Considering the P_ID_ values of the loci analyzed in this study (Table 4), a minimum of 7 loci (from the most to the least informative) is needed to meet the P_IDsib_ threshold of < 0.001, as suggested by Waits et al. [37], while only 5 loci are needed to meet the threshold of < 0.05 used by Woods et al. [20] (Fig. 3). However, in consideration of ABB low variability, these numbers of loci are definitely too low. Waits et al. [37] provided guidelines for researchers computing the theoretical P_ID_ values expected for different heterozygosities and the number of loci that are necessary to achieve a reasonably low P_ID_ (Additional file 7: Fig. S1). This allows to take into account population variability. The value of He of the ABB based on the complete set of 13 STRs (0.464 ± 0.038) corresponds to an approximate minimum number of 11 loci. Regarding P_IDsib_, the number of loci required is between 20 and 25: this value can be considered as a conservative upper bound. Another criterion to establish a threshold for P_IDsib_ is to take into account the dimension of the population. The last estimate of the population size is 50 bears [52], hence a threshold of < 0.006 is acceptable considering that it corresponds to 6 individuals with the same multilocus genotype out of 1000 (0.3 individuals in a population of 50 bears).

Since monitoring of the ABB population has been inconstant over time, it is not fully clear whether the population is decreasing or stable. According to the last population size estimates [41, 50–52] the number of bears is about 40–50 individuals and appears to be stable [55, 67]. However, little is known about the genetic diversity and how it changed over time. Here we provided an estimate of the variation in genetic diversity over time by dividing in two periods the known genotypes, using as a divider the start of the Life *Arctos* project in 2011. That year was chosen because it represents a stop in the genetic monitoring of the core area by the Lab3 and the beginning of genetic monitoring for the Lab2, to compare the results of before and after the Life *Arctos* project. Given that the population has been persisting in small numbers for decades, it is expected that genetic variability has suffered because of genetic drift. The differences in allelic patterns are not significant as showed by the overlap of the error bars and confirmed by the PCA analysis.

Moreover, when the genotypes were analyzed all together, locus Mu11 showed the possible presence of null alleles because of an excess of homozygosis, and a non significant deviation from Hardy-Weinberg equilibrium (Table 3). When the genotypes were split in two time periods (pre-Arctos and Arctos & post-Arctos) locus Mu11 is again in HWE suggesting the possible presence of null alleles only in the second period. A possible explanation is that this locus shows the effect of genetic drift on genotypic frequencies, having therefore an excess of homozygosis or, more probably, a sampling bias since the monitoring of the core area was not constant over time.

As the Apennine brown bear is an iconic taxon of great interest for conservation but its extinction risk is still unknown, our data will provide a tool to choose the best strategy to manage and protect this population. We believe the implications of our results extend beyond the particular species and population we are dealing with. In particular, they are relevant for small populations, whose detectability and genetic variability are expected to be low.

## Conclusions

The identification of core microsatellite panels, detailed analysis protocols, and workflows, represent important steps towards improving the repeatability and comparison of results obtained from different genetic studies on the same species. Although many researchers, to ensure the comparability of their results, select the panel to use based on the markers analyzed in previous studies, it is still common to use panels of different loci even to study the same species in the same geographical area. Accurate evaluation of commonly used markers and the adoption of a shared panel would improve the ability to compare the outcome among studies.

In addition to the optimal STR panel, consisting of 11 *Ursus* specific markers plus cxx20 (the canid-locus less prone to genotyping errors), REN144A06 (H_e_ = 0.605) can be used to improve the discriminatory capacity in case of invasive samples, but should not be used for routine analysis of non-invasive samples due to the high occurrence of genotyping errors.

Non-invasive sampling allows the identification of individuals through DNA fingerprinting techniques and it is the first step to capture-mark-recapture studies to assess the size of natural populations. However, it is difficult to obtain accurate estimates using the low quantities of DNA that can be extracted from non-invasive samples like hairs and feces [10, 37]. The use of panels of short STRs markers (< 150 bp) is the key for successful genotyping of degraded DNA. Indeed, a robust amplification of degraded DNA by short markers improves genotyping success and error rates, particularly for non-invasive samples, increasing detection with limiting amount of template [35]. Therefore it is important to choose the optimal set of STR markers, with low molecular weight, high discriminatory power and low occurrence of genotyping errors.

Accordingly, the proposed marker set:

1. owns a high discriminatory power (P_ID_ = 8.6 × 10^− 6^; P_IDsib_ = 3.0 × 10^− 3^) that prevents underestimation of the number of individuals, according to the thresholds proposed by Waits et al. [37] and Woods et al. [20] and confirmed by the low number of genotypes mismatching at 0, 1 and 2 loci;
2. shows a low chance of genotyping errors (mean ADO = 0.043; mean FA = 0.005) that prevents overestimation of the number of individuals;
3. allows saving time and costs with the amplification in multiplex of all loci thanks to the same annealing temperature of 52.2 °C.

Moreover, future work using non-invasive sampling will also require a rigorous laboratory protocol to minimize genotyping errors. In this study we provide reliable laboratory protocols to deal with non-invasive sampling to assure the quality of the results (Fig. 1).

The identification of shared marker panels facilitates the standardization of the results obtained by different research groups, which in this way can improve their collaboration and conservation efforts.

Despite the Apennine brown bears genetic variability seems to be stable, it is likely that it will decrease in the future, especially if the illegal killings do not stop. Therefore, efforts are needed to develop more variable markers with a better discriminatory capacity. Such markers can be specific single-nucleotide polymorphisms (SNPs), designed on the Apennine brown bear genome [45]. In fact, SNPs can be used in high numbers and open more lines of research such as the estimation of kinships.

## Methods

### Literature review

We performed a literature search for articles published between 2004 and 2017 that utilize STR markers to assess ABB genetics. We recorded the STR loci used in each study. Studies were then sorted by STR loci used and application to determine the potential utility of these markers for common genetic analyses. Following the same logic of the study described in Gervasi et al. [55], we focused on the two labs that have most recently provided the genetic analysis of ABB, to ensure comparability among individual bears sampled over time.

### Samples collection and selection

We sampled 125 different wild bears living in the Abruzzo Lazio and Molise National Park (PNALM) ecosystem in 20 years of monitoring, 12 exclusively through blood sampling of bears live-captured and released for research purposes [50], 7 exclusively through tissue samples extracted from bears found dead and 86 exclusively through non-invasive sampling, by remote collection of tufts of hairs or feces deposited by bears, during population surveys [52]; the remaining 20 bears were sampled both invasively and non-invasively. The majority of individual bears included in our study, therefore, have been systematically or opportunistically sampled from the wild Apennine brown bear population using non-invasive methods [41]. Assuming a relatively stable population of about 50 bears [55], we sampled 125 different bears during the years of our study (from 2000 to 2020), which we considered largely representative both for the selection and the calibration of the genetic marker set.

To date, more than 3500 DNA samples (with associated biological samples belonging to 125 different Apennine bears collected from 2000 to 2020) are stored in the twenty-year Italian national genetic ABB biobank held by ISPRA BIO-CGE.

For calibrating allele calls and verify matches among genotypes obtained in the different studies, six invasive DNA samples from six different individuals were selected from those analyzed during the Life Arctos Project ([52] Additional file 1: Table S1). These six invasive samples were selected ad hoc to represent all alleles present in the ABB population at all tested loci.

For evaluating the microsatellite panels, two DNA samples that produced good quality data from previous STR analysis [14, 41, 50, 54] were selected, when available in the national genetic ABB biobank, for each of the 125 known bears. We prioritized DNA extracted from blood or tissue samples, then from non-invasive systematic sampling and non-invasive opportunistic samples, with more recent samples preferred to older samples and hair preferred to feces. Fecal samples were the last choice.

### Genetic analyses

In order to assure the reliability of future monitoring avoiding excessive genotyping efforts, we selected, based on the bibliographic research carried out, the complete set of STRs to test (Table 1), including firstly all the European brown bear loci used, secondly the most used loci among the American black bear loci and, finally, the canid loci used during the Life Arctos Project.

To perform the calibration and allow the comparison among datasets, six invasive DNA samples (Additional file 1: Table S1) were amplified with the complete set of selected STRs. The results obtained were compared with those obtained, on the same DNA samples, by the lab involved in the Life Arctos Project and the conversion factors (differences in the allele calls in terms of base pair — bp) for each allele present in the ABB population were reconstructed.

To evaluate the marker polymorphism and power, we used the complete set of STRs to reanalyze all the selected DNA samples (two DNA samples, mainly non-invasive samples, for each known bear, when available) and then we identified 4 sets of STRs to compare (see the Results chapter, paragraph: Review of microsatellite and panel selection).

Invasive and non-invasive genomic DNA was extracted in a dedicated room within a sterile UV hood (hood that includes ultraviolet lights for sterilization), using QIAGEN DNeasy® Blood & Tissue Kit (QIAGEN Hilden, Germany), according to the manufacturer’s protocol, using a robotic workstation for automated purification of DNA (QIAcube HT96 platform, QIAGEN - Hilden, Germany).

For the invasive samples, we used the following simplex polymerase chain reaction (PCR) protocol: each locus was amplified separately in a volume of 8 μl containing about 20 DNA nanograms, 1.5 mM MgCl_2_, 0.8 μl of 0.2% BSA, 0.20 μl of each 10 μM primer solution, 0.4 μl of 10 mM dNTPs, 0.2 units of Taq polymerase (Eppendorf), and the following thermal profiles: pre-denaturation at 94 °C for 2 min, followed by 55 cycles with denaturation at 94 °C for 15 s, primer specific annealing temperature (Ta = 52.5 °C for the ursid loci, Ta = 57 °C for the canid loci) for 30 s and extension at 72 °C for 30 s, followed by a final extension at 72 °C for 10 min.

For the non-invasive samples, instead, since the DNA obtained from non-invasive sampling is often diluted and deteriorated, a multiple tubes approach was used to prevent stochastic errors [10]: multilocus genotypes were obtained evaluating the results of four independent PCR replicates. We optimized PCR conditions for use with the QIAGEN® Multiplex PCR Kit (QIAGEN Hilden, Germany). Microsatellite primers were multiplexed on the basis of annealing temperature, allele size distribution, dye color, and stutter pattern to warranty efficiency and minimize genotyping errors. A total of three PCR multiplexes were identified (Table 3). STR amplification was performed using PCR in 8 μl total reaction volumes, containing: 3.5 μl 2x QIAGEN Multiplex PCR Master Mix (providing a final concentration of 3 mM MgCl_2_), 0.7 μl 5x Q-solution (an additive that enables efficient amplification of difficult templates — e.g., GC-rich), multiplex primer cocktail with a total volume equal to the sum of individual primer volumes (10 μM), 2 μl of 10–20 ng/μl DNA template, and deionized H_2_O to 8 μl. Thermal cycling conditions were adapted from the manufacturer’s recommended conditions: 95 °C for 15 min, 45 cycles of 95 °C for 30 s, primer specific annealing temperature (Ta = 52.5/57 °C) for 90 s, and 72 °C for 1 min, followed by a final extension of 72 °C for 10 min.

All PCRs were prepared under a sterile airflow hood cleaned with UV light and processed on Mastercycler® pro S (Eppendorf) and Veriti™ 96-Well Thermal Cycler (Applied Biosystems™). Negative controls were used during each step from DNA extractions to PCR. Positive controls (samples with known genotypes) were added to each PCR session. One primer of each pair was 5′-labeled with 6-FAM, HEX, NED or PET dyes. STR fragments were detected and sized relative to GeneScan™ 500 LIZ® Size Standard (Applied Biosystems™) through capillary electrophoresis in a separate room on an ABI Prism 3130XL Genetic Analyzer DNA sequencer (Thermo Fisher Scientific, Waltham, MA USA) at the ISPRA BIO-CGE lab. The electropherograms were collected by the Data Collection Software v.3.0 and analysed by the GeneMapper® Software v.4.0 (Applied Biosystems by Thermo Fisher Scientific).

### Data analysis

Firstly, once the conversion factors were obtained, we converted the genotypes identified during the Life Arctos project to compare them with the genotypes stored in the Italian national genetic ABB biobank held by ISPRA BIO-CGE (and vice versa) using GenAlEx v6.5 [68] (from the “Multilocus” menu, selecting “Matches”), in order to evaluate the consistency of the data obtained from different laboratories on the same animals, using sets of markers partially different.

Secondly, as a measure of data quality, and in order to select a panel that avoids the overestimation of the population size, we estimated genotyping error rates (ADO, FA, null alleles) using GIMLET v1.3.3 [69] and MicroChecker v2.2.3 [70]. The reliability of each genotype was determined using RelioType [53], with a confidence level of 95%, following the procedure illustrated in Fig. 1.

In order to evaluate the optimal set of STR markers to use in long-term monitoring projects, we compared data from 4 different STR marker subsets (see Additional file 2: Table S2), on the basis of allelic patterns, observed (H_o_) and expected (H_e_) heterozygosity, number of mismatching pairs, P_ID_, and P_IDsib_. The software Popgene v1.32 [71] was used to estimate the variability of allelic patterns of each STR (number of polymorphic loci; total, observed and effective number of alleles; Hardy-Weinberg Equilibrium; H_o_ and H_e_; and Shannon information index). The discriminatory power of every subset of markers is compared using GenAlEx v6.5 and the allelematch R package [72] by calculating the P_ID_, the probability of identity among siblings, the number of 0, 1, 2, and 3 MM among genotypes, and the power in individual identification. The P_ID_ and P_IDsib_ values were computed for one-to-all available loci, by adding loci sequentially, in order from the highest to the lowest level of informativeness, based on the expected number of different individuals with the same genotype at a given locus (GenAlEx v6.5). The values obtained are compared to the P_ID_ threshold proposed by Waits et al. [37] for forensic genetics in wildlife studies (P_ID_ < 0.001) and to the P_IDsib_ threshold used by Woods et al. [20] for brown bear population estimation from hair samples (P_IDsib_ < 0.05). The probability of mismatches was evaluated with the software MM-DIST [73], which calculates probability distributions for the number of loci that individuals in a population will differ by. Summary statistics for each marker locus (including allele number, missing proportion, heterozygosity) were estimated by PowerMarker v3.25 [74] to evaluate the discriminatory power of different primers. The statistical significance of the differences found among different subsets was examined by using the Chi-Square test and the Fisher test in R software (R Development Core Team). *P* values lower than 0.001 were considered statistically significant.

The optimal markers set was selected evaluating the low molecular weight, the high discriminatory power, and the low occurrence of genotyping errors of each primer.

To evaluate changes in population genetic variability over time, allelic patterns of the complete set of selected STRs were compared by dividing the genotypes listed in the national reference database into two subgroups (from 2000 to 2010, *N* = 62 pop1 - pre-arctos and from 2011 to 2017, *N* = 36 pop2 - arctos & post-arctos). The genotypes that were sampled in both periods (*N* = 14) were eliminated from the analysis to avoid their flattening effect on the results. That way, the two time periods comprise two generations each. The two groups of genotypes were analyzed with GenAlEx v6.5 comparing mean allelic patterns across populations (from the “Frequency” menu, selecting “Allelic Pattern Options”). A PCA was performed with the R package “pca3d” [75] and statistical confidence was tested with an AMOVA test. F statistics were calculated for each locus and averaged with FSTAT 2.9.3.2 [76] that also performed jackknifing over loci.

## Supplementary Information

**Additional file 1: Table S1.** List of invasive samples used to verify matches among genotypes and calculate scores’ calibration. List of the 6 selected samples representing 6 genetically different individuals sharing the lowest number of alleles, therefore resuming the observed variability of the whole data set. These samples were analyzed by Lab2 during the Life Arctos Project [52] and by Lab3 during the present study, in order to verify matches among genotypes and calculate scores’ calibration between labs.

**Additional file 2: Table S2.** Four different STR markers sets compared in this study. Both labs use the Amelogenin gene (AMG) to assess gender. ^a^ [52], ^b^ [14, 41, 50].

**Additional file 3: Table S3.** PCR primer sequences used at Lab2, at Lab3 and in this study for the Apennine brown bear individual identification.

**Additional file 4: Table S4.** Putative identical genotypes that differ between labs. Genotypes ram0587 and HS374 were identified by Lab2, genotypes Gen 108 and Gen 105 were identified by Lab3. ^a^ missing data, ^b^ mismatched loci.

**Additional file 5: Table S5.** List of 194 selected samples representing 115 individual bear genotypes out of 125 (ISPRA BIO-CGE). When available, samples from invasive (blood and tissues) and systematic sampling (hairs) were preferred to non-invasive and opportunistic ones, the more recent to the older samples, hairs to feces. Since samples from 10 genotypes (Italian national reference biobank, ISPRA BIO-CGE) were not available, their genotypes were not updated with CXX20 and REN144A06 loci

**Additional file 6: Table S6.** Sampling trend of Apennine brown bear individuals over times. Genotypes were subdivided into two groups based on the years of sampling: 2000–2010 pre-arctos bears, 2011–2017 arctos and post arctos bears. Individuals sampled in both periods were eliminated from the analysis.

**Additional file 7: Fig. S1.** Relationship between theoretical probability of identity and number of loci assayed using four heterozygosity levels. (a) randomly sampled individuals and (b) sibs. From [37].

**Additional file 8: Table S7.** Allelic patterns in 2000–2010 (pop1 - pre-arctos) and 2011–2017 (pop2 - arctos & post arctos). *Na* number of different alleles, *Na Freq. ≥5%* number of alleles with a frequency ≥ 5%, *Ne* number of effective alleles, *I* Shannon Information Index, *No. Private Alleles* number of private alleles, *H*_*o*_ observed heterozygosity and *H*_*e*_ expected heterozygosity.

## Data Availability

The microsatellite genotypes generated and analyzed during the current study are not publicly available due to ongoing work on the genetics of these populations but are available from the corresponding author on reasonable request.

## Abbreviations

STR(s): Short Tandem Repeat(s)
NIGS: Non-invasive genetic sampling
QC: Quality-control
ABB: Apennine brown bear
PATOM: Action plan for the conservation of ABB
CMR: Capture-mark-recapture
IZSAM: Experimental Zooprophylactic Institute of Abruzzo and Molise (Lab1, Method 1)
WGI: Wildlife Genetics International lab (Lab2, Method 2)
ISPRA BIO-CGE: Unit for Conservation Genetics of the Italian Institute for Environmental Protection and Research (Lab3, Method 3)
HWE: Hardy–Weinberg equilibrium
ADO: Allelic dropouts
FA: False alleles
P_ID_: Probability of identity
P_IDsib_: Probability of identity among siblings
MM: Mismatches
PCA: Principal component analysis
AMOVA: Analysis of molecular variance
SNP(s): Single-nucleotide polymorphism(s)
PNALM: Abruzzo Lazio and Molise National Park
UV hood: Hood that includes ultraviolet lights for sterilization
PCR: Polymerase chain reaction
