# Supplementary material for: First core microsatellite panel identification in Apennine brown bears (Ursus arctos marsicanus): a collaborative approach
Source: BMC Genomics. 2021 Aug 18;22:623. doi: 10.1186/s12864-021-07915-5 (PMC8371798; doi:10.1186/s12864-021-07915-5)
Supplement: Supplementary file 1 — Additional file 1: Table S1. List of invasive samples used to verify matches among genotypes and calculate scores’ calibration. List of the 6 selected samples representing 6 genetically different individuals sharing the lowest number of alleles, therefore resuming the observed variability of the whole data set. These samples were analyzed by Lab2 during the Life Arctos Project [52] and by Lab3 during the present study, in order to verify matches among genotypes and calculate scores’ calibration between labs. [file 12864_2021_7915_MOESM1_ESM.docx]

**Additional file 1: Table S1.** List of invasive samples used to verify matches among genotypes and calculate scores' calibration.

| Bear ID  (Lab2) | Bear ID  (Lab3) | Name of bear | Sex | Biological sample | Sampling method |
| --- | --- | --- | --- | --- | --- |
| M10 | Gen 10 | Ciccio | M | Blood tampon | Invasive |
| F04 | Gen 12 | Orsa Maggiore | F | Blood tampon | Invasive |
| M12 | Gen 24 | Cicerone | M | Blood tampon | Invasive |
| M07 | Gen 45 | Edoardo | M | Blood tampon | Invasive |
| M08 | Gen 60 | Stefano | M | Blood tampon | Invasive |
| F09 | Gen 74 | Forchetta | F | Blood tampon | Invasive |

List of the 6 selected samples representing 6 genetically different individuals sharing the lowest number of alleles, resuming the observed variability of the whole data set. These samples were analyzed by Lab2 during the Life Arctos Project [52] and by Lab3 during the present study, in order to calculate scores' calibration between labs and verify matches among 45 genotypes of individuals, that overlapped between Labs 2 and 3.
