# Supplementary material for: First core microsatellite panel identification in Apennine brown bears (Ursus arctos marsicanus): a collaborative approach
Source: BMC Genomics. 2021 Aug 18;22:623. doi: 10.1186/s12864-021-07915-5 (PMC8371798; doi:10.1186/s12864-021-07915-5)
Supplement: Supplementary file 2 — Additional file 2: Table S2. Four different STR markers sets compared in this study. Both labs use the Amelogenin gene (AMG) to assess gender. a [52], b [14, 41, 50]. [file 12864_2021_7915_MOESM2_ESM.docx]

**Additional file 2: Table S2.** Four different STR markers sets compared in this study.

| Locus | Loci  Lab2^a^ | Loci  Lab3^b^ | Loci shared  between labs | Complete set  of 13 loci |
| --- | --- | --- | --- | --- |
| CXX20 | + |  |  | + |
| REN144A06 | + |  |  | + |
| G1D | + | + | + | + |
| Mu51 | + | + | + | + |
| G10B | + | + | + | + |
| G10C | + | + | + | + |
| Mu59 | + | + | + | + |
| Mu11 | + | + | + | + |
| Mu05 | + | + | + | + |
| G10L | + | + | + | + |
| Mu50 | + | + | + | + |
| G10P |  | + |  | + |
| Mu15 |  | + |  | + |
| Total | 11 | 11 | 9 | 13 |

Both labs use the Amelogenin gene (AMG) to assess gender. ^a^ [52], ^b^ [14, 41, 50].
