# Supplementary material for: First core microsatellite panel identification in Apennine brown bears (Ursus arctos marsicanus): a collaborative approach
Source: BMC Genomics. 2021 Aug 18;22:623. doi: 10.1186/s12864-021-07915-5 (PMC8371798; doi:10.1186/s12864-021-07915-5)
Supplement: Supplementary file 3 — Additional file 3: Table S3. PCR primer sequences used at Lab2, at Lab3 and in this study for the Apennine brown bear individual identification. [file 12864_2021_7915_MOESM3_ESM.docx]

**Additional file 3: Table S3.** PCR primer sequences used at Lab2, at Lab3 and in this study for the Apennine brown bear individual identification.

| Primer  Lab2 | Sequences  Lab2 | References | Primer  Lab3 | Sequences  Lab3 and this study | References |
| --- | --- | --- | --- | --- | --- |
| SE47 | 5’-CAGCCAAACCTCCCTCTGC-3’ | Ennis and Gallagher (1994) | Amel4 | 5’-AGAGGCAGGTCAGGAAGCAT-3’ | Modified at Lab3 |
| SE48 | 5’-CCCGCTTGGTCTTGTCTGTTGC-3’ | Ennis and Gallagher (1994) | SE47 | 5’-CAGCCAAACCTCCCTCTGC-3’ | Ennis and Gallagher (1994) |
| CXX20_F | 5’-AGCAACCCCTCCCATTTACT-3’ | [66] | CXX20_F | 5’-AGCAACCCCTCCCATTTACT-3’ | [66] |
| CXX20_R | 5’-TTGTCTGAATAGTCCTCTGCG-3’ | [66] | CXX20_R | 5’-TTGTCTGAATAGTCCTCTGCG-3’ | [66] |
| REN144A06_F | 5’-TTTTATGGTTGAGTGCTATTCC-3’ | [65] | REN144A06_F | 5’-TTTTATGGTTGAGTGCTATTCC-3’ | [65] |
| REN144A06_R | 5’-GAAATTGGCCACAGTTCCAT-3’ | [65] | REN144A06_R | 5’-GAAATTGGCCACAGTTCCAT-3’ | [65] |
| G1D_F | 5’-ATCTGTGGGTTTATAGGTTACA-3’ | [21] | G1D_FIm | 5’-CCATCTCTCTTTTCCTTTAGGG-3’ | Modified at Lab3 |
| G1D_R | 5’-CTAGCACCCAGCAAGGTA-3’ | [21] | G1D_RI | 5’-CTACTCTTCCTACTCTTTAAGAG-3’ | [21] (Internal primer) |
| Mu51_F | 5’-GCCAGAATCCTAAGAGACCT-3’ | [21] | Mu51_Fb | 5’-AGCCAGAATCCTAAGAGACCT-3’ | Modified at Lab3 |
| Mu51_R | 5’-GAAAGGTTAGATGGAAGAGATG-3’ | [21] | Mu51_RIb | 5’-AAAGAGAAGGGACAGGAGGTA-3’ | Modified at Lab3 |
| G10B_F | 5’-AAGCCTTTTAATGTTCTGTTG-3’ | [21] | G10B_FI | 5’-TGCTAATATTTTCTTGAGGACT-3’ | [21] (Internal primer) |
| G10B_R | 5’-AGGACAAATCACAGAAACCT-3’ | [21] | G10B_R | 5’-AGGACAAATCACAGAAACCT-3’ | [21] |
| G10C_F | 5’-CAACAAAAGGTTGAAGGGAG-3’ | [21] | G10C_FI | 5’-GTCTGCAAAAGCAGAAGG-3’ | [21] (Internal primer) |
| G10C_R | 5’-AAACACCGAGACAGCAGG-3’ | [21] | G10C_R | 5’-AAACACCGAGACAGCAGG-3’ | [21] |
| Mu59_F | 5’-GCTCCTTTGGGACATTGTAA-3’ | [21] | Mu59_F | 5’-GCTCCTTTGGGACATTGTAA-3’ | [21] |
| Mu59_R | 5’-TGGATAGCATTCAGGCAT-3’ | [21] | Mu59_RIb | 5’-TGACTGTCACCAGCAGGAG-3’ | Modified at Lab3 |
| Mu11_F | 5’-AATGTGAAAAAGAAAAGGTAGG-3’ | [21] | Mu11_FI | 5’-AAGTAATTGGTGAAATGACAGG-3’ | [21] (Internal primer) |
| Mu11_R | 5’-GAACCCTTCACCGAAAATC-3’ | [21] | Mu11_R | 5’-GAACCCTTCACCGAAAATC-3’ | [21] |
| Mu05_F | 5’-AATCTTTTCACTTATGCCCA-3’ | [21] | Mu05_FI | 5’-AATCTTTTCACTTATGCCCA-3’ | [21] (Internal primer) |
| Mu05_R | 5’-GAAACTTGTTATGGGAACCA-3’ | [21] | Mu05_R | 5’-GAAACTTGTTATGGGAACCA-3’ | [21] |
| G10L_F | 5’-GGACAGGATATTGACATTGA-3’ | [21] | G10L_FI | 5’-ACTGATTTTATTCACATTTCCC-3’ | [21] (Internal primer) |
| G10L_R | 5’-CAGAAACCTACCCATGCG-3’ | [21] | G10L_R | 5’-GATACAGAAACCTACCCATGCG-3’ | Modified at Lab3 |
| Mu50_F | 5’-TCTCTGTCATTTCCCCATC-3’ | [21] | Mu50_Fb | 5’-GTCTCTGTCATTTCCCCATC-3’ | Modified at Lab3 |
| Mu50_R | 5’-AAAGGCAATGCAGATATTGT-3’ | [21] | Mu50_RIb | 5’-AACCTGGAACAAAAATTAACAC-3’ | Modified at Lab3 |
| G10P_F | 5’-CCAGGGCAAGAAATAATGAG-3’ | [21] | G10P_Fp | 5’-AGTTTTACATAGGAGGAAGAA-3’ | Modified at Lab3 |
| G10P_R | 5’-AAAAGGCCTAAGCTACATCG-3’ | [21] | G10P_Rp | 5’-TCATGTGGGGAAATACTCTGAA-3’ | Modified at Lab3 |
| Mu15_F | Not used at Lab2 | [21] | Mu15_FI | 5’-CTGAATTATGCAATTAAACAGC-3’ | [21] (Internal primer) |
| Mu15_R | Not used at Lab2 | [21] | Mu15_R | 5’-AAATAAGGGAGGCTTGGGT-3’ | [21] |
