# Supplementary material for: First core microsatellite panel identification in Apennine brown bears (Ursus arctos marsicanus): a collaborative approach
Source: BMC Genomics. 2021 Aug 18;22:623. doi: 10.1186/s12864-021-07915-5 (PMC8371798; doi:10.1186/s12864-021-07915-5)
Supplement: Supplementary file 4 — Additional file 4: Table S4. Putative identical genotypes that differ between labs. Genotypes ram0587 and HS374 were identified by Lab2, genotypes Gen 108 and Gen 105 were identified by Lab3. a missing data, b mismatched loci. [file 12864_2021_7915_MOESM4_ESM.docx]

**Additional file 4: Table S4.** Putative identical genotypes that differ between labs.

| Couples of  genotypes | G10B | G10C | G10L | G10P | G1D | Mu05 | Mu11 | Mu15 | Mu50 | Mu51 | Mu59 | CXX20 | REN144  A06 |
| --- | --- | --- | --- | --- | --- | --- | --- | --- | --- | --- | --- | --- | --- |
| Pair 1 |  |  |  |  |  |  |  |  |  |  |  |  |  |
| ram0587 | 112/112 | 101/105 | 148/154 | 0/0^a^ | 100/114 | 135/137 | 88/92 | 0/0 ^a^ | 100/104 | 114/114 | 101/101 | 136/136 ^b^ | 128/128 ^b^ |
| Gen 108 | 112/112 | 101/105 | 148/154 | 164/164 | 100/114 | 135/137 | 88/92 | 121/121 | 100/104 | 114/114 | 101/101 | 134/134 ^b^ | 110/110 ^b^ |
| Pair 2 |  |  |  |  |  |  |  |  |  |  |  |  |  |
| HS374 | 112/128 | 101/105 | 154/154 | 164/164 | 100/114 | 135/137 | 92/92 | 0/0 ^a^ | 100/100 | 114/120 | 101/107 | 134/134 ^b^ | 128/128 ^b^ |
| Gen 105 | 112/128 | 101/105 | 154/154 | 164/164 | 100/114 | 135/137 | 92/92 | 121/121 | 100/100 | 114/120 | 101/107 | 132/134 ^b^ | 110/128 ^b^ |

Genotypes ram0587 and HS374 were identified by Lab2, genotypes Gen 108 and Gen 105 were identified by Lab3. ^a^ missing data, ^b^ mismatched loci.
