# Supplementary material for: First core microsatellite panel identification in Apennine brown bears (Ursus arctos marsicanus): a collaborative approach
Source: BMC Genomics. 2021 Aug 18;22:623. doi: 10.1186/s12864-021-07915-5 (PMC8371798; doi:10.1186/s12864-021-07915-5)
Supplement: Supplementary file 5 — Additional file 5: Table S5. List of 194 selected samples representing 115 individual bear genotypes out of 125 (ISPRA BIO-CGE). When available, samples from invasive (blood and tissues) and systematic sampling (hairs) were preferred to non-invasive and opportunistic ones, the more recent to the older samples, hairs to feces. Since samples from 10 genotypes (Italian national reference biobank, ISPRA BIO-CGE) were not available, their genotypes were not updated with CXX20 and REN144A06 loci [file 12864_2021_7915_MOESM5_ESM.docx]

**Additional file 5: Table S5.** List of 194 selected samples representing 115 individual bear genotypes out of 125 (ISPRA BIO-CGE - Lab3).

| Bear ID | Sample ID | Name of bear | Sex | Year | Sampling method | Biological sample | Notes |
| --- | --- | --- | --- | --- | --- | --- | --- |
| Gen 2 | OA0735 |  | F | 2004 | Non invasive | Hairs |  |
| Gen 2 | OA0736 |  | F | 2004 | Non invasive | Hairs |  |
| Gen 3 | OA0030 |  | M | 2000 | Non invasive | Hairs |  |
| Gen 3 | OA0032 |  | M | 2000 | Non invasive | Hairs |  |
| Gen 4 | OA1540 |  | F | 2007 | Non invasive | Hairs |  |
| Gen 4 | OA1542 |  | F | 2007 | Non invasive | Hairs |  |
| Gen 5 | OA0541 |  | M | 2003 | Non invasive | Hairs |  |
| Gen 5 | OA0553 |  | M | 2003 | Non invasive | Hairs |  |
| Gen 6 | OA0067 |  | M | 2001 | Non invasive | Hairs |  |
| Gen 6 | OA0068 |  | M | 2001 | Non invasive | Hairs |  |
| Gen 7 | OA1657 | Gemma | F | 2008 | Non invasive | Hairs |  |
| Gen 7 | OA1660 | Gemma | F | 2008 | Non invasive | Hairs |  |
| Gen 9 | OA1791 | Claudio/Renato | M | 2008 | Non invasive | Hairs |  |
| Gen 9 | OA1793 | Claudio/Renato | M | 2008 | Non invasive | Hairs |  |
| Gen 10 | OA1423 | Ciccio | M | 2005 | Non invasive | Hairs |  |
| Gen 10 | OA1424 | Ciccio | M | 2005 | Non invasive | Hairs |  |
| Gen 11 | OA1430 | Bernardo | M | 2005 | Non invasive | Hairs |  |
| Gen 11 | OA1815 | Bernardo | M | 2008 | Invasive | Blood |  |
| Gen 12 | OA1667 | Orsa Maggiore | F | 2008 | Non invasive | Hairs |  |
| Gen 12 | OA1687 | Orsa Maggiore | F | 2008 | Non invasive | Hairs |  |
| Gen 13 | OA0192 |  | F | 2002 | Non invasive | Faeces |  |
| Gen 13 | OA0251 |  | F | 2002 | Non invasive | Faeces |  |
| Gen 16 | OA0131 |  | Ind | 2002 | Non invasive | Faeces |  |
| Gen 18 | OA1762 |  | F | 2008 | Non invasive | Hairs |  |
| Gen 18 | OA2716 |  | F | 2016 | Invasive | Blood |  |
| Gen 19 | OA1739 |  | F | 2008 | Non invasive | Hairs | 3 alleles at REN144A06 |
| Gen 19 | OA1740 |  | F | 2008 | Non invasive | Hairs | 3 alleles at REN144A06 |
| Gen 20 | OA1823 |  | M | 2008 | Invasive | Blood |  |
| Gen 20 | OA1892 |  | M | 2010 | Invasive | Blood |  |
| Gen 21 | OA1413 | Nestore | M | 2005 | Non invasive | Hairs |  |
| Gen 21 | OA1804 | Nestore | M | 2008 | Invasive | Blood |  |
| Gen 22 | OA1545 | Atessa | F | 2007 | Non invasive | Hairs |  |
| Gen 22 | OA1812 | Atessa | F | 2008 | Invasive | Blood |  |
| Gen 23 | OA1799 | Ura | F | 2008 | Invasive | Blood |  |
| Gen 23 | OA1817 | Ura | F | 2008 | Invasive | Blood |  |
| Gen 24 | OA1689 | Cicerone | M | 2008 | Non invasive | Hairs |  |
| Gen 24 | OA1808 | Cicerone | M | 2008 | Invasive | Blood |  |
| Gen 25 | OA1419 | Reginella | F | 2005 | Non invasive | Hairs | 3 alleles at REN144A06 |
| Gen 25 | OA1807 | Reginella | F | 2008 | Invasive | Blood |  |
| Gen 28 | OA0386 |  | Ind | 2003 | Non invasive | Hairs |  |
| Gen 29 | OA0449 |  | M | 2003 | Non invasive | Faeces |  |
| Gen 31 | OA1362 | Tranquilla | F | 2005 | Non invasive | Hairs |  |
| Gen 31 | OA1890 | Tranquilla | F | 2010 | Invasive | Blood |  |
| Gen 32 | OA1291 |  | F | 2005 | Non invasive | Hairs |  |
| Gen 32 | OA1426 |  | F | 2005 | Non invasive | Hairs |  |
| Gen 33 | OA1544 |  | F | 2007 | Non invasive | Hairs |  |
| Gen 33 | OA1880 |  | F | 2009 | Invasive | Tissue |  |
| Gen 34 | OA0715 |  | F | 2004 | Non invasive | Hairs |  |
| Gen 34 | OA0716 |  | F | 2004 | Non invasive | Hairs |  |
| Gen 35 | OA0515 |  | M | 2003 | Non invasive | Faeces |  |
| Gen 36 | OA0767 |  | F | 2004 | Non invasive | Hairs |  |
| Gen 36 | OA1028 |  | F | 2005 | Non invasive | Hairs |  |
| Gen 37 | OA1289 |  | F | 2005 | Non invasive | Hairs |  |
| Gen 37 | OA1510 |  | F | 2007 | Non invasive | Hairs |  |
| Gen 38 | OA1299 |  | F | 2005 | Non invasive | Hairs |  |
| Gen 38 | OA1318 |  | F | 2005 | Non invasive | Hairs |  |
| Gen 40 | OA0599 |  | M | 2003 | Non invasive | Hairs |  |
| Gen 41 | OA1398 |  | F | 2005 | Non invasive | Hairs |  |
| Gen 41 | OA1399 |  | F | 2005 | Non invasive | Hairs |  |
| Gen 43 | OA1653 |  | F | 2008 | Non invasive | Hairs |  |
| Gen 43 | OA1654 |  | F | 2008 | Non invasive | Hairs |  |
| Gen 44 | OA1676 | Valery | F | 2008 | Non invasive | Hairs | 3 alleles at REN144A06 |
| Gen 44 | OA1809 | Valery | F | 2008 | Invasive | Blood |  |
| Gen 45 | OA1443 | Edoardo | M | 2005 | Non invasive | Hairs |  |
| Gen 45 | OA1806 | Edoardo | M | 2008 | Invasive | Blood |  |
| Gen 46 | OA1382 |  | M | 2005 | Non invasive | Hairs |  |
| Gen 46 | OA1383 |  | M | 2005 | Non invasive | Hairs |  |
| Gen 47 | OA0763 |  | F | 2004 | Non invasive | Hairs |  |
| Gen 48 | OA0765 |  | F | 2004 | Non invasive | Hairs |  |
| Gen 49 | OA1724 |  | M | 2008 | Non invasive | Hairs |  |
| Gen 49 | OA1746 |  | M | 2008 | Non invasive | Hairs |  |
| Gen 50 | OA2775 | Monachella | F | 2017 | Non invasive | Hairs |  |
| Gen 50 | OA2814 | Monachella | F | 2017 | Invasive | Blood |  |
| Gen 51 | OA1453 | Perrone | M | 2006 | Non invasive | Hairs |  |
| Gen 51 | OA1600 | Perrone | M | 2008 | Non invasive | Hairs |  |
| Gen 52 | OA0947 |  | M | 2004 | Non invasive | Hairs | 3 alleles at REN144A06 |
| Gen 53 | OA0981 |  | F | 2004 | Non invasive | Hairs | Removed afterwards (see text) |
| Gen 53 | OA1357 |  | F | 2005 | Non invasive | Hairs | 3 alleles at CXX20 and REN144A06  Removed afterwards (see text) |
| Gen 54 | OA1818 | Marina | F | 2004 | Invasive | Blood |  |
| Gen 54 | OA1801 | Marina | F | 2008 | Invasive | Blood |  |
| Gen 55 | OA0992 |  | F | 2004 | Non invasive | Hairs | 3 alleles at CXX20 and REN144A06 |
| Gen 56 | OA1436 | Stella | F | 2005 | Non invasive | Hairs |  |
| Gen 56 | OA1805 | Stella | F | 2008 | Invasive | Blood |  |
| Gen 57 | OA1414 | Ilaria | F | 2005 | Non invasive | Hairs |  |
| Gen 57 | OA1813 | Ilaria | F | 2008 | Invasive | Blood |  |
| Gen 58 | OA1397 |  | F | 2005 | Non invasive | Hairs |  |
| Gen 58 | OA1593 |  | F | 2008 | Non invasive | Hairs |  |
| Gen 59 | OA1697 |  | F | 2008 | Non invasive | Hairs |  |
| Gen 59 | OA2797 |  | F | 2017 | Non invasive | Hairs |  |
| Gen 60 | OA1794 | Stefano | M | 2008 | Non invasive | Hairs |  |
| Gen 60 | OA1816 | Stefano | M | 2008 | Invasive | Blood |  |
| Gen 61 | OA1249 |  | M | 2005 | Non invasive | Hairs |  |
| Gen 61 | OA1461 |  | M | 2006 | Non invasive | Hairs |  |
| Gen 62 | OA1208 |  | M | 2005 | Non invasive | Hairs |  |
| Gen 62 | OA1240 |  | M | 2005 | Non invasive | Hairs |  |
| Gen 63 | OA1237 |  | F | 2005 | Non invasive | Hairs |  |
| Gen 63 | OA1238 |  | F | 2005 | Non invasive | Hairs |  |
| Gen 64 | OA1323 |  | M | 2005 | Non invasive | Hairs |  |
| Gen 64 | OA1324 |  | M | 2005 | Non invasive | Hairs |  |
| Gen 65 | OA1447 |  | M | 2005 | Non invasive | Hairs |  |
| Gen 65 | OA1872 |  | M | 2008 | Invasive | Hairs + tissue |  |
| Gen 66 | OA2712 | Vittorio | M | 2013 | Invasive | Blood |  |
| Gen 66 | OA2732 | Vittorio | M | 2016 | Non invasive | Hairs |  |
| Gen 67 | OA1255 |  | F | 2005 | Non invasive | Hairs |  |
| Gen 68 | OA1374 |  | M | 2005 | Non invasive | Hairs |  |
| Gen 69 | OA1321 |  | M | 2005 | Non invasive | Hairs |  |
| Gen 70 | OA1650 | Ulisse | M | 2007 | Non invasive | Hairs |  |
| Gen 70 | OA1901 | Ulisse | M | 2009 | Non invasive | Hairs |  |
| Gen 71 | OA1681 | Tommaso | M | 2008 | Non invasive | Hairs |  |
| Gen 71 | OA1759 | Tommaso | M | 2008 | Non invasive | Hairs | 3 alleles at REN144A06 |
| Gen 72 | OA1749 | Ferroio | M | 2008 | Non invasive | Hairs |  |
| Gen 72 | OA1824 | Ferroio | M | 2008 | Invasive | Blood |  |
| Gen 73 | OA1825 | Sebastiana | F | 2008 | Invasive | Blood |  |
| Gen 74 | OA1826 | Forchetta | F | 2008 | Invasive | Blood |  |
| Gen 74 | OA1888 | Forchetta | F | 2010 | Invasive | Blood |  |
| Gen 75 | OA1828 | Silvia | F | 2008 | Invasive | Blood |  |
| Gen 76 | OA2786 |  | M | 2017 | Non invasive | Hairs |  |
| Gen 76 | OA2796 |  | M | 2017 | Non invasive | Hairs |  |
| Gen 77 | OA1881 |  | M | 2007 | Invasive | Tissue |  |
| Gen 78 | OA1882 |  | F | 2007 | Invasive | Tissue |  |
| Gen 79 | OA1883 |  | F | 2006 | Invasive | Tissue |  |
| Gen 80 | OA1886 |  | F | 2008 | Invasive | Tissue |  |
| Gen 81 | OA1887 | Faustino | M | 2010 | Invasive | Blood |  |
| Gen 81 | OA2810 | Faustino | M | 2017 | Non invasive | Hairs |  |
| Gen 82 | OA1889 | Vincenzina | F | 2010 | Invasive | Blood |  |
| Gen 83 | OA1891 | Fortunato | M | 2010 | Invasive | Blood |  |
| Gen 84 | OA2165 |  | F | 2012 | Non invasive | Hairs |  |
| Gen 84 | OA2351 |  | F | 2011 | Non invasive | Hairs | Bear-canid mixed sample |
| Gen 85 | OA1992 |  | F | 2010 | Non invasive | Hairs |  |
| Gen 85 | OA2171 |  | F | 2012 | Non invasive | Hairs |  |
| Gen 86 | OA2057 |  | M | 2012 | Non invasive | Hairs |  |
| Gen 86 | OA2182 |  | M | 2012 | Non invasive | Hairs |  |
| Gen 87 | OA1583 |  | M | 2007 | Invasive | Tissue | brother of OA1584 |
| Gen 88 | OA1584 |  | M | 2007 | Invasive | Tissue | brother of OA1583 |
| Gen 89 | OA2079 |  | M | 2012 | Non invasive | Faeces |  |
| Gen 89 | OA2080 |  | M | 2012 | Non invasive | Faeces |  |
| Gen 90 | OA2090 |  | F | 2011 | Non invasive | Hairs |  |
| Gen 90 | OA2093 |  | F | 2011 | Non invasive | Hairs |  |
| Gen 91 | OA2186 |  | M | 2012 | Non invasive | Hairs |  |
| Gen 91 | OA2203 |  | M | 2013 | Non invasive | Hairs |  |
| Gen 92 | OA2106 |  | F | 2012 | Non invasive | Hairs |  |
| Gen 92 | OA2107 |  | F | 2012 | Non invasive | Hairs |  |
| Gen 93 | OA2556 |  | M | 2015 | Non invasive | Hairs |  |
| Gen 93 | OA2567 |  | M | 2015 | Non invasive | Hairs |  |
| Gen 94 | OA2266 |  | M | 2013 | Non invasive | Hairs |  |
| Gen 95 | OA2410 |  | M | 2014 | Non invasive | Hairs |  |
| Gen 95 | OA2480 |  | M | 2014 | Non invasive | Hairs |  |
| Gen 96 | OA2040 |  | F | 2012 | Non invasive | Hairs |  |
| Gen 97 | OA2395 |  | M | 2014 | Non invasive | Hairs |  |
| Gen 97 | OA2414 |  | M | 2014 | Invasive | Hairs |  |
| Gen 98 | OA2396 |  | M | 2014 | Non invasive | Hairs | Removed afterwards (see text) |
| Gen 98 | OA2404 |  | M | 2014 | Non invasive | Hairs | Removed afterwards (see text) |
| Gen 99 | OA2713 | Peppina | F | 2015 | Invasive | Blood |  |
| Gen 99 | OA2746 | Peppina | F | 2017 | Non invasive | Hairs |  |
| Gen 99 | OA2819 | Peppina | F | 2017 | Non invasive | Hairs |  |
| Gen 100 | OA2622 |  | F | 2016 | Non invasive | Hairs |  |
| Gen 100 | OA2774 |  | F | 2017 | Non invasive | Hairs |  |
| Gen 101 | OA2595 |  | F | 2016 | Non invasive | Hairs |  |
| Gen 101 | OA2695 |  | F | 2016 | Non invasive | Hairs |  |
| Gen 102 | OA2490 | Morena | F | 2015 | Invasive | Hairs |  |
| Gen 103 | OA2527 |  | F | 2015 | Non invasive | Hairs |  |
| Gen 103 | OA2538 |  | F | 2015 | Non invasive | Hairs |  |
| Gen 104 | OA2555 |  | M | 2015 | Non invasive | Hairs |  |
| Gen 105 | OA2625 |  | M | 2016 | Non invasive | Hairs |  |
| Gen 105 | OA2739 |  | M | 2017 | Non invasive | Hairs |  |
| Gen 106 | OA2764 |  | M | 2017 | Non invasive | Hairs |  |
| Gen 106 | OA2820 |  | M | 2017 | Non invasive | Hairs |  |
| Gen 107 | OA2617 |  | M | 2016 | Non invasive | Hairs |  |
| Gen 107 | OA2620 |  | M | 2016 | Non invasive | Hairs |  |
| Gen 108 | OA2612 |  | M | 2016 | Non invasive | Hairs |  |
| Gen 109 | OA2633 |  | M | 2016 | Non invasive | Hairs |  |
| Gen 109 | OA2639 |  | M | 2016 | Non invasive | Hairs |  |
| Gen 110 | OA2665 |  | M | 2016 | Non invasive | Hairs |  |
| Gen 111 | OA2758 |  | M | 2017 | Non invasive | Hairs |  |
| Gen 111 | OA2759 |  | M | 2017 | Non invasive | Hairs |  |
| Gen 112 | OA2684 |  | M | 2015 | Non invasive | Hairs |  |
| Gen 113 | OA2727 | Mario | M | 2017 | Non invasive | Hairs |  |
| Gen 113 | OA2805 | Mario | M | 2017 | Non invasive | Hairs |  |
| Gen 114 | OA2745 |  | M | 2017 | Non invasive | Hairs |  |
| Gen 114 | OA2807 |  | M | 2017 | Non invasive | Hairs |  |
| Gen 115 | OA2717 |  | F | 2016 | Invasive | Blood |  |
| Gen 116 | OA2737 |  | M | 2017 | Non invasive | Hairs |  |
| Gen 116 | OA2751 |  | M | 2017 | Non invasive | Hairs |  |
| Gen 117 | OA2714 |  | M | 2016 | Invasive | Blood |  |
| Gen 118 | OA2718 |  | F | 2016 | Invasive | Blood |  |
| Gen 119 | OA2705 |  | F | 2016 | Non invasive | Hairs |  |
| Gen 119 | OA2724 |  | F | 2017 | Non invasive | Hairs |  |
| Gen 120 | OA2741 |  | M | 2017 | Non invasive | Hairs |  |
| Gen 121 | OA2707 |  | F | 2016 | Non invasive | Hairs |  |
| Gen 122 | OA2794 |  | M | 2017 | Non invasive | Hairs |  |
| Gen 122 | OA2798 |  | M | 2017 | Non invasive | Hairs |  |
| Gen 123 | OA2799 |  | F | 2017 | Non invasive | Hairs |  |
| Gen 124 | OA2800 |  | F | 2017 | Non invasive | Hairs |  |
| Gen 125 | OA2813 | Libera | F | 2017 | Invasive | Blood |  |

When available, samples from invasive (blood and tissues) and systematic sampling (hairs) were preferred to non-invasive and opportunistic ones, the more recent to the older samples, hairs to feces. Since samples from 10 genotypes (Italian national reference biobank, ISPRA BIO-CGE) were not available, their genotypes were not updated with CXX20 and REN144A06 loci.
