# Supplementary material for: First core microsatellite panel identification in Apennine brown bears (Ursus arctos marsicanus): a collaborative approach
Source: BMC Genomics. 2021 Aug 18;22:623. doi: 10.1186/s12864-021-07915-5 (PMC8371798; doi:10.1186/s12864-021-07915-5)
Supplement: Supplementary file 6 — Additional file 6: Table S6. Sampling trend of Apennine brown bear individuals over times. Genotypes were subdivided into two groups based on the years of sampling: 2000–2010 pre-arctos bears, 2011–2017 arctos and post arctos bears. Individuals sampled in both periods were eliminated from the analysis. [file 12864_2021_7915_MOESM6_ESM.docx]

**Additional file 6: Table S6.** Sampling trend of Apennine brown bear individuals over times.

|  |  | pop1 - pre-arctos | | | | | | | | | | | pop2 - arctos&post | | | | | | |
| --- | --- | --- | --- | --- | --- | --- | --- | --- | --- | --- | --- | --- | --- | --- | --- | --- | --- | --- | --- |
| ID |  | 2000 | 2001 | 2002 | 2003 | 2004 | 2005 | 2006 | 2007 | 2008 | 2009 | 2010 | 2011 | 2012 | 2013 | 2014 | 2015 | 2016 | 2017 |
| N MALES-M |  | 4 | 2 | 9 | 8 | 7 | 18 | 7 | 9 | 14 | 3 | 7 | 2 | 4 | 6 | 4 | 6 | 13 | 12 |
| N FEMALES-F |  | 3 | 4 | 9 | 14 | 24 | 24 | 2 | 11 | 20 | 3 | 7 | 3 | 6 | 3 | 6 | 7 | 11 | 9 |
| TOT | Sex | 7 | 6 | 18 | 22 | 31 | 42 | 9 | 20 | 34 | 6 | 14 | 5 | 10 | 9 | 10 | 13 | 24 | 21 |
| Gen1.2 | F | X |  |  |  | X |  |  |  |  |  |  |  |  |  |  |  |  |  |
| Gen1.3 | M | X | X | X |  |  |  |  |  |  |  |  |  |  |  |  |  |  |  |
| Gen1.4 | F | X |  | X | X | X | X |  | X | X | X |  |  |  |  |  |  |  |  |
| Gen1.5 | M | X |  | X | X |  |  |  |  |  |  |  |  |  |  |  |  |  |  |
| Gen1.6 | M | X | X | X |  |  |  |  |  |  |  |  |  |  |  |  |  |  |  |
| Gen1.7 | F | X | X | X | X | X | X | X | X | X | X |  |  |  |  | X |  |  |  |
| Gen1.8 | F |  | X |  |  |  |  |  |  |  |  |  |  |  |  |  |  |  |  |
| Gen1.9 | M | X |  | X |  |  | X | X | X | X |  |  |  |  |  |  |  |  |  |
| Gen1.10 | M |  |  | X | X | X | X |  |  |  |  |  |  |  |  |  |  |  |  |
| Gen1.11 | M |  |  | X | X | X | X | X |  | X |  |  |  |  |  |  |  |  |  |
| Gen1.12 | F |  | X | X | X | X | X |  |  | X |  |  |  |  |  |  |  |  |  |
| Gen1.13 | F |  | X | X |  |  |  |  |  |  |  |  |  |  |  |  |  |  |  |
| Gen1.18 | F |  |  | X | X | X | X |  | X | X |  |  |  |  |  |  |  | X |  |
| Gen1.19 | F |  |  | X |  | X | X |  | X | X |  |  |  |  |  |  |  |  |  |
| Gen1.20 | M |  |  | X |  |  |  |  | X | X |  | X |  |  |  |  |  |  |  |
| Gen1.21 | M |  |  | X | X |  | X |  |  | X |  |  |  |  |  |  |  |  |  |
| Gen1.22 | F |  |  | X |  |  |  |  | X | X |  |  |  |  |  |  |  |  |  |
| Gen1.23 | F |  |  | X | X | X | X |  | X | X |  |  |  |  |  |  | X |  |  |
| Gen1.24 | M |  |  | X | X |  | X |  | X | X |  |  |  |  |  |  |  |  |  |
| Gen1.25 | F |  |  | X | X | X | X |  |  | X |  |  |  |  |  |  |  |  |  |
| Gen1.29 | M |  |  |  | X |  |  |  |  |  |  |  |  |  |  |  |  |  |  |
| Gen1.31 | F |  |  |  | X | X | X |  | X |  |  | X |  |  |  |  |  |  |  |
| Gen1.32 | F |  |  |  | X | X | X |  |  |  |  |  |  |  |  |  |  |  |  |
| Gen1.33 | F |  |  |  | X | X | X |  | X |  | X |  |  |  |  |  |  |  |  |
| Gen1.34 | F |  |  |  | X | X |  |  |  |  |  |  |  |  |  |  |  |  |  |
| Gen1.35 | M |  |  |  | X |  |  |  |  |  |  |  |  |  |  |  |  |  |  |
| Gen1.36 | F |  |  |  | X | X | X |  |  |  |  |  |  |  |  |  |  |  |  |
| Gen1.37 | F |  |  |  | X | X | X |  | X |  |  |  |  |  |  |  |  |  |  |
| Gen1.38 | F |  |  |  | X | X | X |  |  |  |  |  |  |  |  |  |  |  |  |
| Gen1.40 | M |  |  |  | X |  |  |  |  |  |  |  |  |  |  |  |  |  |  |
| Gen1.41 | F |  |  |  | X | X | X |  |  |  |  | X |  |  | X |  | X | X | X |
| Gen1.43 | F |  |  |  |  | X | X |  |  | X |  |  |  |  | X |  |  |  |  |
| Gen1.44 | F |  |  |  |  | X | X |  |  | X |  |  |  |  |  |  |  |  |  |
| Gen1.45 | M |  |  |  |  | X | X |  |  | X |  |  |  |  |  |  |  |  |  |
| Gen1.46 | M |  |  |  |  | X | X |  |  |  |  |  |  |  |  |  |  |  |  |
| Gen1.47 | F |  |  |  |  | X |  |  |  |  |  |  |  |  |  |  |  |  |  |
| Gen1.48 | F |  |  |  |  | X |  |  |  |  |  |  |  |  |  |  |  |  |  |
| Gen1.49 | M |  |  |  |  | X | X |  |  | X |  | X |  |  |  |  |  |  |  |
| Gen1.50 | F |  |  |  |  | X | X |  |  | X |  |  |  | X |  | X |  | X | X |
| Gen1.51 | M |  |  |  |  | X | X | X |  | X |  |  |  |  |  |  |  |  |  |
| Gen1.52 | M |  |  |  |  | X |  |  |  |  |  |  |  |  |  |  |  |  |  |
| Gen1.54 | F |  |  |  |  | X | X |  |  | X |  |  |  |  |  |  |  |  |  |
| Gen1.55 | F |  |  |  |  | X |  |  |  |  |  |  |  |  |  |  |  |  |  |
| Gen1.56 | F |  |  |  |  | X | X |  |  | X |  |  |  |  |  |  |  |  |  |
| Gen1.57 | F |  |  |  |  |  | X |  |  | X |  |  |  |  |  |  |  |  |  |
| Gen1.58 | F |  |  |  |  |  | X |  |  | X |  |  |  |  |  |  |  |  |  |
| Gen1.59 | F |  |  |  |  |  | X |  | X | X |  | X | X | X | X | X | X | X | X |
| Gen1.60 | M |  |  |  |  |  | X |  | X | X |  |  |  |  |  |  |  |  |  |
| Gen1.61 | M |  |  |  |  |  | X | X |  |  |  |  |  |  |  |  |  |  |  |
| Gen1.62 | M |  |  |  |  |  | X |  |  |  |  |  |  |  |  |  |  |  |  |
| Gen1.63 | F |  |  |  |  |  | X |  |  |  |  |  |  |  |  |  |  |  |  |
| Gen1.64 | M |  |  |  |  |  | X |  |  |  |  |  |  |  |  |  |  |  |  |
| Gen1.65 | M |  |  |  |  |  | X | X |  | X |  |  |  |  |  |  |  |  |  |
| Gen1.66 | M |  |  |  |  |  | X |  |  | X |  |  |  |  | X |  |  | X | X |
| Gen1.67 | F |  |  |  |  |  | X |  |  |  |  |  |  |  |  |  |  |  |  |
| Gen1.68 | M |  |  |  |  |  | X |  |  |  |  |  |  |  |  |  |  |  |  |
| Gen1.69 | M |  |  |  |  |  | X |  |  |  |  |  |  |  |  |  |  |  |  |
| Gen1.70 | M |  |  |  |  |  |  | X | X | X | X | X |  |  |  |  |  |  |  |
| Gen1.71 | M |  |  |  |  |  | X |  | X | X |  |  |  |  |  |  |  |  |  |
| Gen1.72 | M |  |  |  |  |  |  | X |  | X | X | X |  |  | X |  |  |  |  |
| Gen1.73 | F |  |  |  |  |  |  |  |  | X |  |  |  |  |  |  |  |  |  |
| Gen1.74 | F |  |  |  |  |  |  |  |  | X |  | X |  |  |  |  |  |  |  |
| Gen1.75 | F |  |  |  |  |  |  |  |  | X |  |  |  |  |  |  |  |  |  |
| Gen1.76 | M |  |  |  |  |  |  |  |  |  | X |  | X |  |  | X | X | X | X |
| Gen1.77 | M |  |  |  |  |  |  |  | X |  |  |  |  |  |  |  |  |  |  |
| Gen1.78 | F |  |  |  |  |  |  |  | X |  |  |  |  |  |  |  |  |  |  |
| Gen1.79 | F |  |  |  |  |  |  | X |  |  |  |  |  |  |  |  |  |  |  |
| Gen1.80 | F |  |  |  |  |  |  |  |  | X |  |  |  |  |  |  |  |  |  |
| Gen1.81 | M |  |  |  |  |  |  |  |  |  |  | X |  |  |  |  |  |  | X |
| Gen1.82 | F |  |  |  |  |  |  |  |  |  |  | X |  |  |  |  |  |  |  |
| Gen1.83 | M |  |  |  |  |  |  |  |  |  |  | X |  |  |  |  |  |  |  |
| Gen1.84 | F |  |  |  |  |  |  |  |  |  |  | X | X | X |  |  |  |  |  |
| Gen1.85 | F |  |  |  |  |  |  |  |  |  |  | X |  | X |  |  |  |  |  |
| Gen1.86 | M |  |  |  |  |  |  |  |  |  |  | X |  | X |  |  |  |  |  |
| Gen1.87 | M |  |  |  |  |  |  |  | X |  |  |  |  |  |  |  |  |  |  |
| Gen1.88 | M |  |  |  |  |  |  |  | X |  |  |  |  |  |  |  |  |  |  |
| Gen1.89 | M |  |  |  |  |  |  |  |  |  |  |  |  | X |  |  |  |  |  |
| Gen1.90 | F |  |  |  |  |  |  |  |  |  |  |  | X |  |  |  |  |  |  |
| Gen1.91 | M |  |  |  |  |  |  |  |  |  |  |  | X | X | X |  |  |  |  |
| Gen1.92 | F |  |  |  |  |  |  |  |  |  |  |  |  | X |  |  |  |  |  |
| Gen1.93 | M |  |  |  |  |  |  |  |  |  |  |  |  | X | X | X | X |  |  |
| Gen1.94 | M |  |  |  |  |  |  |  |  |  |  |  |  |  | X |  |  |  |  |
| Gen1.95 | M |  |  |  |  |  |  |  |  |  |  |  |  |  | X | X |  |  |  |
| Gen1.96 | F |  |  |  |  |  |  |  |  |  |  |  |  | X |  |  |  |  |  |
| Gen1.97 | M |  |  |  |  |  |  |  |  |  |  |  |  |  |  | X |  |  |  |
| Gen1.99 | F |  |  |  |  |  |  |  |  |  |  |  |  |  |  | X | X | X | X |
| Gen1.100 | F |  |  |  |  |  |  |  |  |  |  |  |  |  |  | X | X | X | X |
| Gen1.101 | F |  |  |  |  |  |  |  |  |  |  |  |  |  |  | X |  | X |  |
| Gen1.102 | F |  |  |  |  |  |  |  |  |  |  |  |  |  |  |  | X |  |  |
| Gen1.103 | F |  |  |  |  |  |  |  |  |  |  |  |  |  |  |  | X |  |  |
| Gen1.104 | M |  |  |  |  |  |  |  |  |  |  |  |  |  |  |  | X |  |  |
| Gen1.105 | M |  |  |  |  |  |  |  |  |  |  |  |  |  |  |  | X | X | X |
| Gen1.106 | M |  |  |  |  |  |  |  |  |  |  |  |  |  |  |  | X | X | X |
| Gen1.107 | M |  |  |  |  |  |  |  |  |  |  |  |  |  |  |  |  | X | X |
| Gen1.108 | M |  |  |  |  |  |  |  |  |  |  |  |  |  |  |  |  | X |  |
| Gen1.109 | M |  |  |  |  |  |  |  |  |  |  |  |  |  |  |  |  | X |  |
| Gen1.110 | M |  |  |  |  |  |  |  |  |  |  |  |  |  |  |  |  | X |  |
| Gen1.111 | M |  |  |  |  |  |  |  |  |  |  |  |  |  |  |  |  | X | X |
| Gen1.112 | M |  |  |  |  |  |  |  |  |  |  |  |  |  |  |  | X |  |  |
| Gen1.113 | M |  |  |  |  |  |  |  |  |  |  |  |  |  |  |  |  | X | X |
| Gen1.114 | M |  |  |  |  |  |  |  |  |  |  |  |  |  |  |  |  | X | X |
| Gen1.115 | F |  |  |  |  |  |  |  |  |  |  |  |  |  |  |  |  | X |  |
| Gen1.116 | M |  |  |  |  |  |  |  |  |  |  |  |  |  |  |  |  | X | X |
| Gen1.117 | M |  |  |  |  |  |  |  |  |  |  |  |  |  |  |  |  | X |  |
| Gen1.118 | F |  |  |  |  |  |  |  |  |  |  |  |  |  |  |  |  | X |  |
| Gen1.119 | F |  |  |  |  |  |  |  |  |  |  |  |  |  |  |  |  | X | X |
| Gen1.120 | M |  |  |  |  |  |  |  |  |  |  |  |  |  |  |  |  |  | X |
| Gen1.121 | F |  |  |  |  |  |  |  |  |  |  |  |  |  |  |  |  | X |  |
| Gen1.122 | M |  |  |  |  |  |  |  |  |  |  |  |  |  |  |  |  |  | X |
| Gen1.123 | F |  |  |  |  |  |  |  |  |  |  |  |  |  |  |  |  |  | X |
| Gen1.124 | F |  |  |  |  |  |  |  |  |  |  |  |  |  |  |  |  |  | X |
| Gen1.125 | F |  |  |  |  |  |  |  |  |  |  |  |  |  |  |  |  |  | X |

Genotypes were subdivided into two groups based on the years of sampling: 2000-2010 pre-arctos bears, 2011-2017 arctos and post arctos bears. Individuals sampled in both periods were eliminated from the analysis.
