# Supplementary material for: First core microsatellite panel identification in Apennine brown bears (Ursus arctos marsicanus): a collaborative approach
Source: BMC Genomics. 2021 Aug 18;22:623. doi: 10.1186/s12864-021-07915-5 (PMC8371798; doi:10.1186/s12864-021-07915-5)
Supplement: Supplementary file 7 — Additional file 7: Fig. S1. Relationship between theoretical probability of identity and number of loci assayed using four heterozygosity levels. (a) randomly sampled individuals and (b) sibs. From [37]. [file 12864_2021_7915_MOESM7_ESM.docx]

**Additional file 7: Fig. S1.** Relationship between theoretical probability of identity and number of loci assayed using four heterozygosity levels.


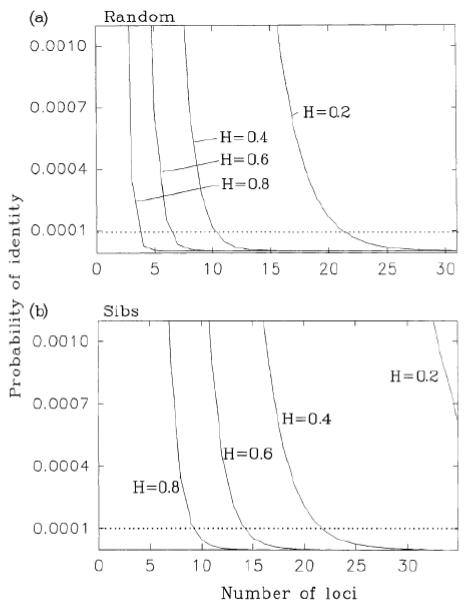


(a) randomly sampled individuals and (b) sibs. From [37].
