# Supplementary material for: First core microsatellite panel identification in Apennine brown bears (Ursus arctos marsicanus): a collaborative approach
Source: BMC Genomics. 2021 Aug 18;22:623. doi: 10.1186/s12864-021-07915-5 (PMC8371798; doi:10.1186/s12864-021-07915-5)
Supplement: Supplementary file 8 — Additional file 8: Table S7. Allelic patterns in 2000–2010 (pop1 - pre-arctos) and 2011–2017 (pop2 - arctos & post arctos). Na number of different alleles, Na Freq. ≥5% number of alleles with a frequency ≥ 5%, Ne number of effective alleles, I Shannon Information Index, No. Private Alleles number of private alleles, Ho observed heterozygosity and He expected heterozygosity. [file 12864_2021_7915_MOESM8_ESM.docx]

**Additional file 8: Table S7.** Allelic patterns in 2000-2010 (pop1 - pre-arctos) and 2011-2017 (pop2 - arctos & post arctos).

|  | pop1  2000-2010  pre-arctos | pop2  2011-2017  arctos&post |
| --- | --- | --- |
| Na | 2.46 ±0.144 | 2.38 ±0.140 |
| Na Freq. >= 5% | 2.30 ±0.133 | 2.30 ±0.133 |
| Ne | 1.96 ±0.104 | 1.92 ±0.125 |
| I | 0.72 ±0.057 | 0.69 ±0.062 |
| No. Private Alleles | 0.15 ±0.104 | 0.07 ±0.077 |
| H_o_ | 0.49 ±0.045 | 0.45 ±0.044 |
| H_e_ | 0.47 ±0.035 | 0.45 ±0.039 |

*Na* number of different alleles, *Na Freq. ≥5%* number of alleles with a frequency ≥5%, *Ne* number of effective alleles, *I* Shannon Information Index, *No. Private Alleles* number of private alleles, *H_o_* observed heterozygosity and *H_e_* expected heterozygosity.
